# Towards Mindless Stress Regulation in Advanced Driver Assistance Systems: A Systematic Review

**DOI:** 10.3389/fpsyg.2020.609124

**Published:** 2020-12-23

**Authors:** Adolphe J. Béquet, Antonio R. Hidalgo-Muñoz, Christophe Jallais

**Affiliations:** ^1^TS2-LESCOT, Univ Gustave Eiffel, IFSTTAR, Univ Lyon, Lyon, France; ^2^CLLE, UMR 5263, CNRS, University of Toulouse Jean-Jaurès, Toulouse, France

**Keywords:** stress, mindless regulation, ADAS (advanced driver assistance system), driving, calm technology, entrainment, biofeeback, affective computing (AC)

## Abstract

**Background:** Stress can frequently occur in the driving context. Its cognitive effects can be deleterious and lead to uncomfortable or risky situations. While stress detection in this context is well developed, regulation using dedicated advanced driver-assistance systems (ADAS) is still emergent.

**Objectives:** This systematic review focuses on stress regulation strategies that can be qualified as “subtle” or “mindless”: the technology employed to perform regulation does not interfere with an ongoing task. The review goal is 2-fold: establishing the state of the art on such technological implementation in the driving context and identifying complementary technologies relying on subtle regulation that could be applied in driving.

**Methods:** A systematic review was conducted using search operators previously identified through a concept analysis. The patents and scientific studies selected provide an overview of actual and potential mindless technology implementations. These are then analyzed from a scientific perspective. A classification of results was performed according to the different stages of emotion regulation proposed by the Gross model.

**Results:** A total of 47 publications were retrieved, including 21 patents and 26 studies. Six of the studies investigated mindless stress regulation in the driving context. Patents implemented strategies mostly linked to attentional deployment, while studies tended to investigate response modulation strategies.

**Conclusions:** This review allowed us to identify several ADAS relying on mindless computing technologies to reduce stress and better understand the underlying mechanisms allowing stress reduction. Further studies are necessary to better grasp the effect of mindless technologies on driving safety. However, we have established the feasibility of their implementation as ADAS and proposed directions for future research in this field.

## Introduction

**Figure 1:**
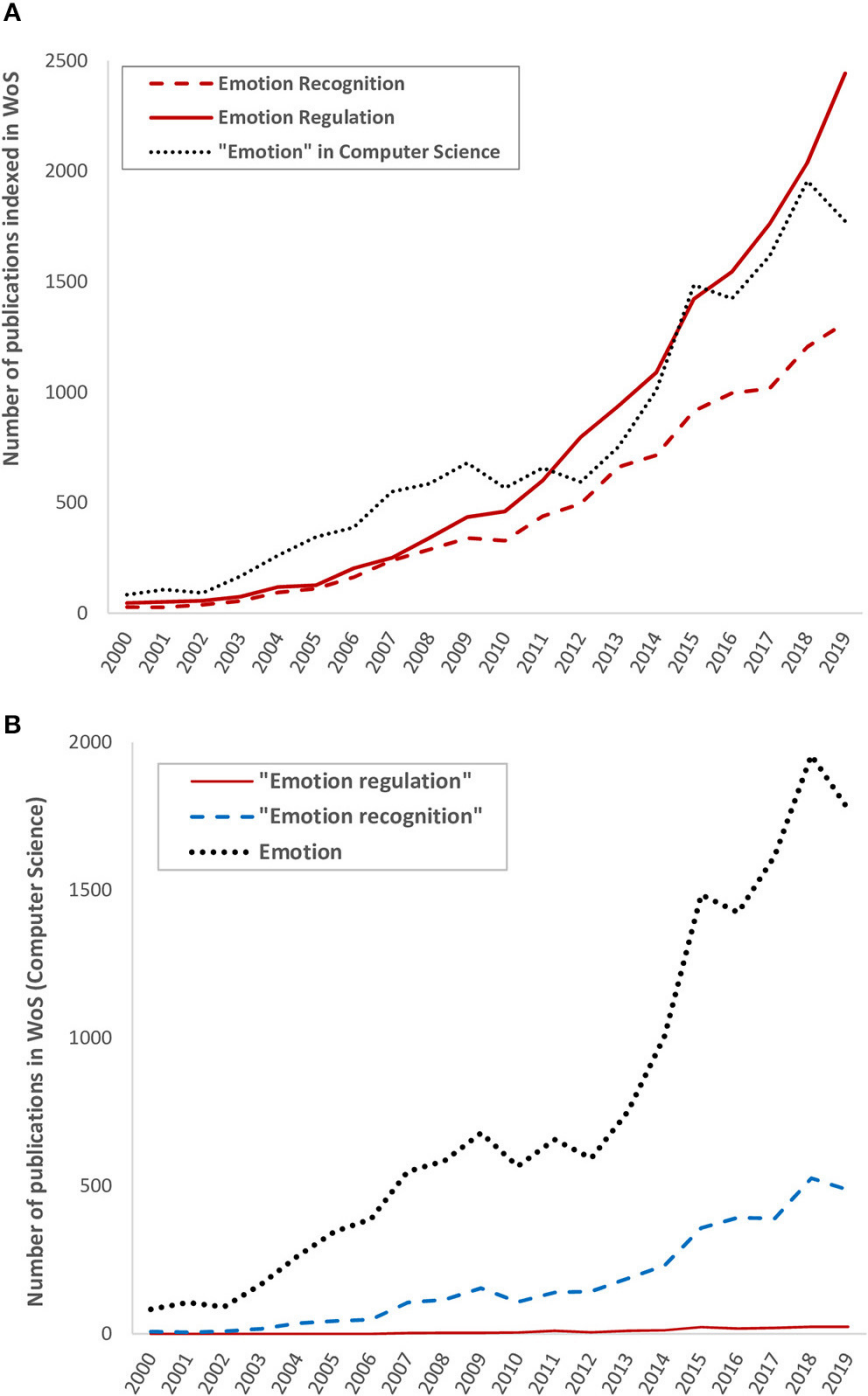
Growing trend of publications in Web of Science since 1990 containing the exact phrases “emotion recognition,” “emotion regulation,” and “emotion” in their abstract and titles for **(A)** all categories of publications indexed in WoS and **(B)** Computer Science publications.

The recent years have seen the rise of advanced driver-assistance systems (ADAS) able to monitor the state of the driver in real time, including or even focusing exclusively on the affective dimension. Recent European projects are funded on this topic^1^^,^^2^. The next step would be to regulate this affective dimension (Zepf et al., 2019). Automotive industrials show a growing interest in this topic (see Braun et al., 2020 for a review). Figure 1 illustrates this tendency. The research concerning “emotion” in computer science, one relevant field for this review, is compared to the interest by other disciplines. As displayed in Figure 1, emotion regulation can be considered a new domain compared to the numerous results for emotion recognition.

Among the different emotions, stress is arguably one of the most interesting to regulate while driving. Stress can be due to environmental stressors as well as personal factors, such as work-related issues (Rowden et al., 2011). Moreover, stress can generate other emotions that impact driver performance, e.g., anger, (Shamoa-Nir and Koslowsky, 2010), or provoke distraction and compromise safety (Brake Direct Line, 2011).

### Stress and Emotional Regulation

Selye (1983) defined stress as “A nonspecific response of the body to any demand” (p. 2). In driving, acute stress can lead to deleterious effects on performance. Of note, this state is different from chronic stress, which impacts people daily without requiring the presence of a specific stressor. Acute stress is time limited and emerges when stressors are present. The possible stressors can be very different [e.g., personal threat, time constraint (Klein, 1996), or cognitive workload (Hidalgo-Muñoz et al., 2019)].

Stress can be physiologically, behaviorally, or psychologically self-regulated (Giannakakis et al., 2019). Under stress, hormones such as noradrenaline or cortisol are released to help the organism adapt to the stressful environment. In high levels of concentration, these hormones can decrease the activity in frontal brain regions (Dehais et al., 2020), regions strongly linked to executive functions and working memory (Motley, 2018), and which support attentional abilities (Dehais et al., 2019). These cognitive functions are involved in event anticipation, the inhibition of distracting stimuli, or mental flexibility (Miyake et al., 2000), which are essential to drive safely.

One of the most accepted models to explain emotional regulation (i.e., the ability to modulate one's own emotions) was proposed by Gross (2014). The author considers the temporal course of emotion emergence to explore five sequential stages where the regulation may occur. These stages are temporally divided depending on whether the emotion is developing or already present. These stages are the following:

- situation selection: avoiding certain situations that could elicit stress;
- situation modification: modifying the stressful situation by acting on stressors;
- attentional deployment: redirecting one's attention away from the stressor;
- cognitive change: re-evaluating the stressful situation (reappraisal);
- response modulation: acting on experiential, behavioral, or physiological components of the stress response.

**Figure 2:**
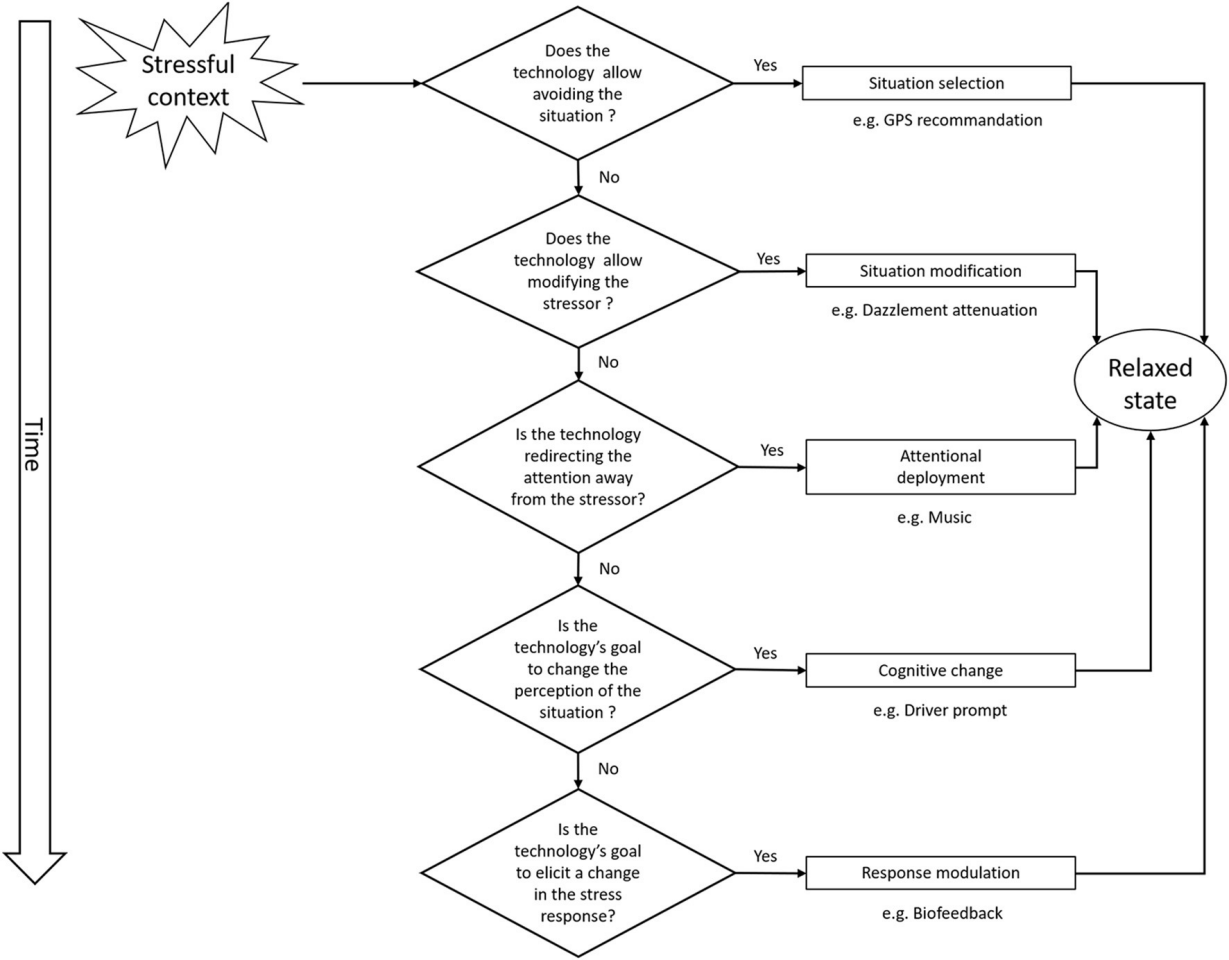
Gross' emotional regulation model adapted decision chart to classify each records according to regulation processes.

Figure 2 presents these stages and some examples of specific techniques that can be deployed in a driving context. Indeed, the selected papers are discussed using this methodology.

### Stress While Driving: Emergence Model, Impacts, and Detection Methods

Driving requires a high level of executive functions, such as decision making, cognitive flexibility, and working memory (Mäntylä et al., 2009; Asimakopulos et al., 2012). Studies have pointed out that stress is commonly felt by drivers. For instance, a report based on a survey of 841 UK drivers (Brake Direct Line, 2011) show that 71% of drivers felt stressed and lost concentration due to stress. Another survey conducted in New Zealand reported that 55% of them reported driver anxiety (Taylor, 2018).

To understand the origins and consequences of stress, Matthews (2002) proposed a transactional model of driver stress and fatigue. Note that, although this review is focused on stress, there are other factors that can impact driving performance and could be considered to regulate stress. Fatigue is an important one (Crawford, 1961). The transactional model presents stress as the product of the driver's evaluation of the environment, mediated by personal coping abilities. In this model, the term “transaction” refers to the relationship between person and environment. This relationship is modulated through cognitive stress processes, linkable to appraisal theories (Scherer, 1999), that allow the person to evaluate the situation. This evaluation is supported by cognitive functions linked to self-consciousness, memory, motivation, reasoning, and attention. The situational demand can be evaluated as either exceeding or being within the limits of the perceived resources of the driver. The outcome of this transaction is expressed in terms of subjective feelings like stress or fatigue and in terms of road behaviors. This transaction is conceived in a dynamic perspective, meaning that a re-evaluation that considers past outcomes can occur, closing the loop. According to appraisal theory (Scherer, 1999), the evaluation is conducted on several dimensions: pertinence (linked to implication), controllability (linked to possible actions), and significance (linked to beliefs) of the situation for the driver.

Situational factors in driving (strains) can induce cognitive stress. These factors include (but are not limited to) traffic congestion, time constraints, and weather (Rowden et al., 2011). Furthermore, the behavior of other drivers can also be an important stressor (Rasmussen et al., 2000).

Concomitant with the situation, several personal factors, e.g., health level, personality traits, or attitudes toward driving, can influence the perception of one's resources. For instance, neuroticism or trait anxiety can increase the negative evaluation of the situation, thus leading to distress (Wang et al., 2018). Furthermore, the occurrence of work-related stress or other life events such as being in the process of a divorce has been shown to be positively correlated with the feeling of stress while driving (Rowden et al., 2011).

As mentioned, stress can reduce cortical activity (Qin et al., 2019), impair executive functions, or even induce attentional tunneling (Dehais et al., 2020). The disruption of these functions can lead to a decrease in situational awareness or to risky behaviors in driving (Healey and Picard, 2005). The question of stress detection and regulation in the car is therefore crucial to ensure safety. However, stress detection should be subtle to avoid disrupting the driving process.

Regarding physiology, various stress indicators derived from autonomic activity are well known: heart rate (HR), breathing rate (BR), or their variability (HRV and BRV, respectively) (Taelman et al., 2009; Vlemincx et al., 2012), or electrodermal activity (EDA) (Setz et al., 2009), among others (see Giannakakis et al., 2019 for a review). Table 1 shows several illustrative examples.

**Table 1:** Examples of studies using biometrics to perform stress detection.

**Study**	**Metrics**	**Subtle detection technique employed**
Kuboi et al. (2016)	Respiration	Ultrawide band radar
Baek et al. (2009)		Piezoelectric sensor in the seatbelt
	Photoplethysmography	LED and photodiodes in the steering wheel
	Galvanic skin response (electrodermal activity)	Copper sheet on the steering wheel
	Electrocardiogram	Electrodes on the seat
Tomii and Ohtsuki (2015)		Ultrawide band radar
Pedrotti et al. (2014)	Pupil diameter	Remote video eye tracker
Gao et al. (2014)	Facial expressions	Near InfraRed camera
Fernandez and Picard (2003)	Voice features analysis	Microphone
Anzengruber and Riener (2012)	Skin temperature	Thermal Infrared Imaging
Beggiato et al. (2018)	Body posture	Seat pressure, motion tracking system

Driving behavior measurements can also be used to detect stress: acceleration, speed patterns (Rastgoo et al., 2019), steering wheel usage (Paredes et al., 2018a), etc. Additionally, the methods can be based on environmental measures (context detection) to determine whether a situation is likely to elicit stress. For example, Vhaduri et al. (2014) managed to detect a driver's stress using GPS traces from a smartphone. In the same vein, Dobbins and Fairclough (2018) used contextual data (photographs of the traffic environment) to improve their stress detection algorithm.

All these stress indicators can be combined to achieve better detection (Abou Elassad et al., 2020).

Although ADAS and driving automatization could reduce the prevalence of stressful and risky situations through the management of some situational stressors (Chung et al., 2019), this is not a full-fledged solution, as it can lead to new forms of stress. This can be due to the attentional solicitation required by their supervision or due to the reduction of situational awareness (Warm et al., 2008). Actually, in some cases, ADAS can act as stressors on board by increasing the mental workload to handle them, increasing the complexity of the cockpit, or through false alarms (Brookhuis et al., 2001). Besides, in autonomous vehicles, drivers can be engaged in nondriving-related tasks and thoughts (Pfleging et al., 2016), which can elicit internal acute stress in various forms. These elements can impact takeover, a procedure that we can expect in near-future automated vehicles, thus inducing new safety challenges (SAE International, 2018).

Stress regulation methods usually imply cognitive therapies, meditation, or respiratory exercises (Baer, 2003), which could turn into cognitive distraction. An interesting approach to design ADAS aiming to regulate stress can lie in the field of “mindless computing,” defined by Adams et al. (2015) as “a mobile or ubiquitous, persuasive technology designed to subtly influence the behavior of the user without requiring their conscious awareness.” Therefore, mindless technologies act on an individual without disturbing him from a main task (i.e., in an unconscious way). As previously illustrated, mindless stress detection can be accomplished using various subtle indicators (e.g., driving context or biosignal remote measurements). Although much less explored, mindless technologies could also be applied for stress regulation, and it is a promising emergent field.

### Aim of the Present Review

The goal of this systematic review is to establish the state of the art in the domain of subtle stress regulation in driving. The works selected will allow better understanding on how this regulation can be technically accomplished and its scientific bases. A second objective is to explore new directions for future research on in-car regulation techniques based on mindless computing. Hence, not only patents and research work investigating it in the driving context (*actual patents and actual papers*) will be considered but also research works that could be applied to driving (*potential papers*).

## Methods

Guidelines and recommendations contained in the Preferred Reporting Items for Systematic Reviews and Meta-analyses (PRISMA) statement (Liberati et al., 2009) have been followed.

### Eligibility Criteria

This review includes articles and patents dealing with mindless computing technologies that aim to regulate acute stress. With this in mind, our eligibility criteria were inspired by Adams et al. (2015) regarding the design considerations of mindless computing technologies:

- Reflexive technologies: the technologies have to leverage prompt and automated cognitive processes.
- Triggering technologies: the desired change of behavior should be achieved without depending on user motivation. Personalization of the trigger per user is possible.
- Parallel technologies: the technology has to be subtle. The user does not have to explicitly attend to it and should be able to pursue another activity without being disturbed by the technology.

We also excluded publications that did not consider stress regulation and focused on other emotions (e.g., anger), or that would use invasive techniques (e.g., use of needles/electrostimulation). Furthermore, we excluded publications that request explicit training from the user to be efficient. This means that studies that would be based on prior implicit learning have been included.

For the first part of this work (*actual* applications developed for driving), patents were favored since they could include the most recent and concrete technical development regarding the automotive industry. We also sought experimental studies that investigate stress regulation in a driving context (simulated or naturalistic).

Regarding the second part of our work (*potential* applications for driving), we based our research on experimental studies and excluded opinion or review papers. We considered studies that focused on adult participants of any age and gender. Studies investigating specific populations as a target to apply the regulation technique on were excluded.

### Information Sources

We used several electronic databases to conduct our search. For the *actual* part, we located patents using Espacenet,^3^ a database inventorying worldwide patent. For the papers (*actual and potential*), we used five electronic databases: Scopus, Web of Science, Association for Computing Machinery Digital Library (ACMDL), PsycInfo, and IEEExplore. We limited our research to English papers and to patents for which an English translation was available on Espacenet. We restricted this search to publication dates from the past decade (2010–2020), since a growing trend of interest toward our research topic seems to appear in this range (Figure 1). When possible, we also limited our search to research fields that would match our review scope to limit the number of false positives.

### Search

To conduct a systematic search, we first identified concepts linked to our research topic in both parts of the review. The concepts included for the *potential* part were the following: “regulation,” “stress,” “mindless,” and “technology.” For the concepts in the *actual* part, the “driving” concept was added to select patents that were specific to the driving context. We chose to exclude the concepts linked to “mindless computing” and “technology” from the patent equation search due to limitations of the search engine and because we stated that emotional regulation while driving manually would, in most cases, be a mindless technique due to the cognitive and attentional requirements of the manual driving context. The screening phases allowed us to include patents that matched this criterion. We excluded patents that focused on nonmindless techniques but included patents that referred to several techniques including mindless and nonmindless techniques.

We then defined various keywords that could be linked to each concept and which were expected to appear in the titles or the abstracts of studies regarding mindless stress regulation. Due to a large number of keywords, we pretested various research equations that could apply to each of the requirements and limitations of the selected database to obtain results that would neither be too restrictive nor too inclusive. Therefore, two main research equations were retained.

For the patents search: [(driver) AND (regulat*OR recov*OR relax*OR modulat* OR manag* OR reduc*) AND (stress OR anxiety OR emotion)].

Given the search restriction for Espacenet, we chose to use the search token “*” to expand our research to all keywords included in the concepts of “regulation” in the equation for the Potential part.

For the studies search: [*(regulate OR regulation OR recover OR recovery OR relax OR relaxation OR modulate OR modulation OR manage OR management OR reduce OR reduction) AND (stress OR anxiety OR emotion) AND (subtle OR implicit OR seamless OR subconscious OR mindless OR peripheral OR nonintrusive) AND (device OR technology OR intervention OR technique OR wearable)*].

### Study and Patent Selection

**Figure 3:**
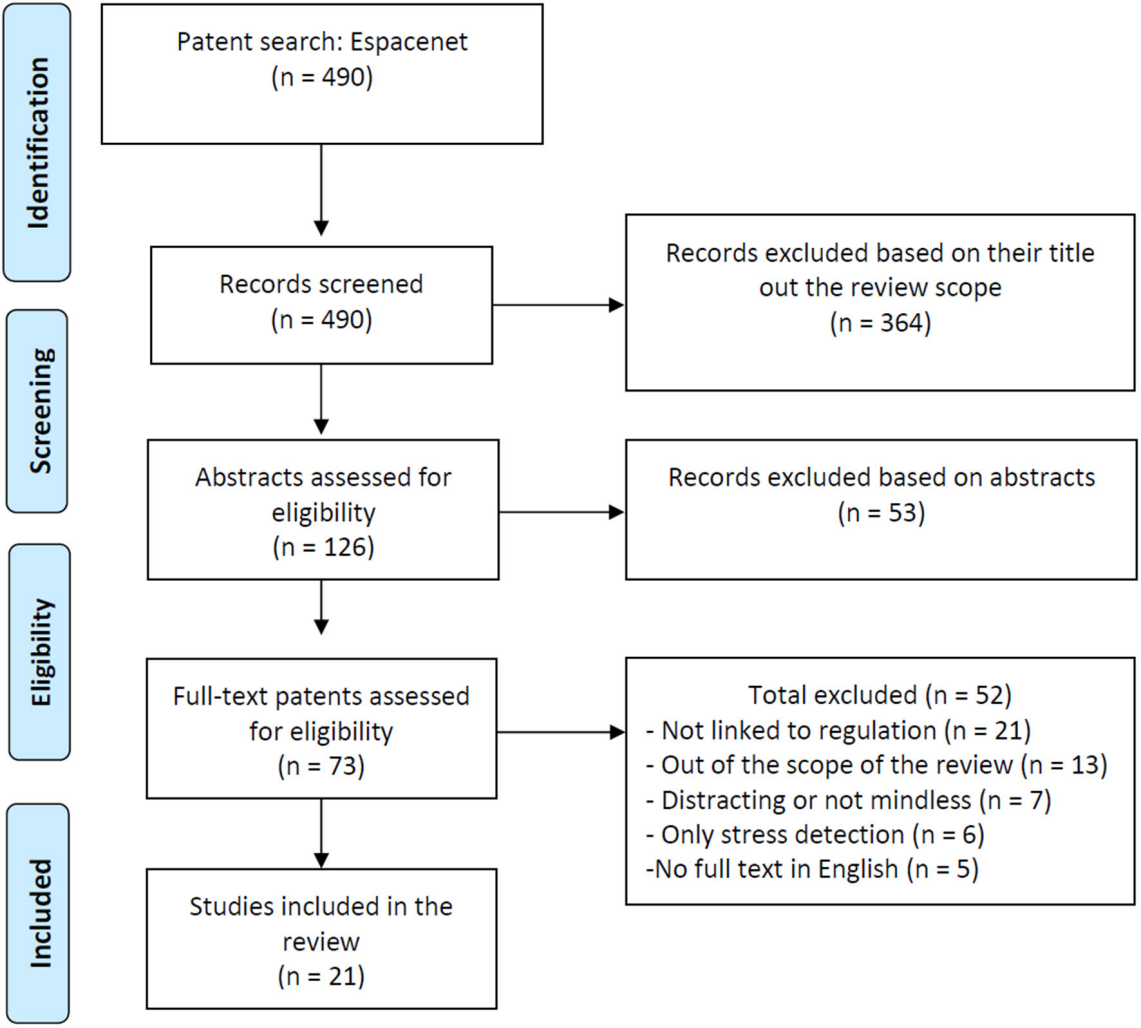
Adapted preferred reporting items for systematic reviews and meta-analyses (PRISMA) flowchart for patent selection.

**Figure 4:**
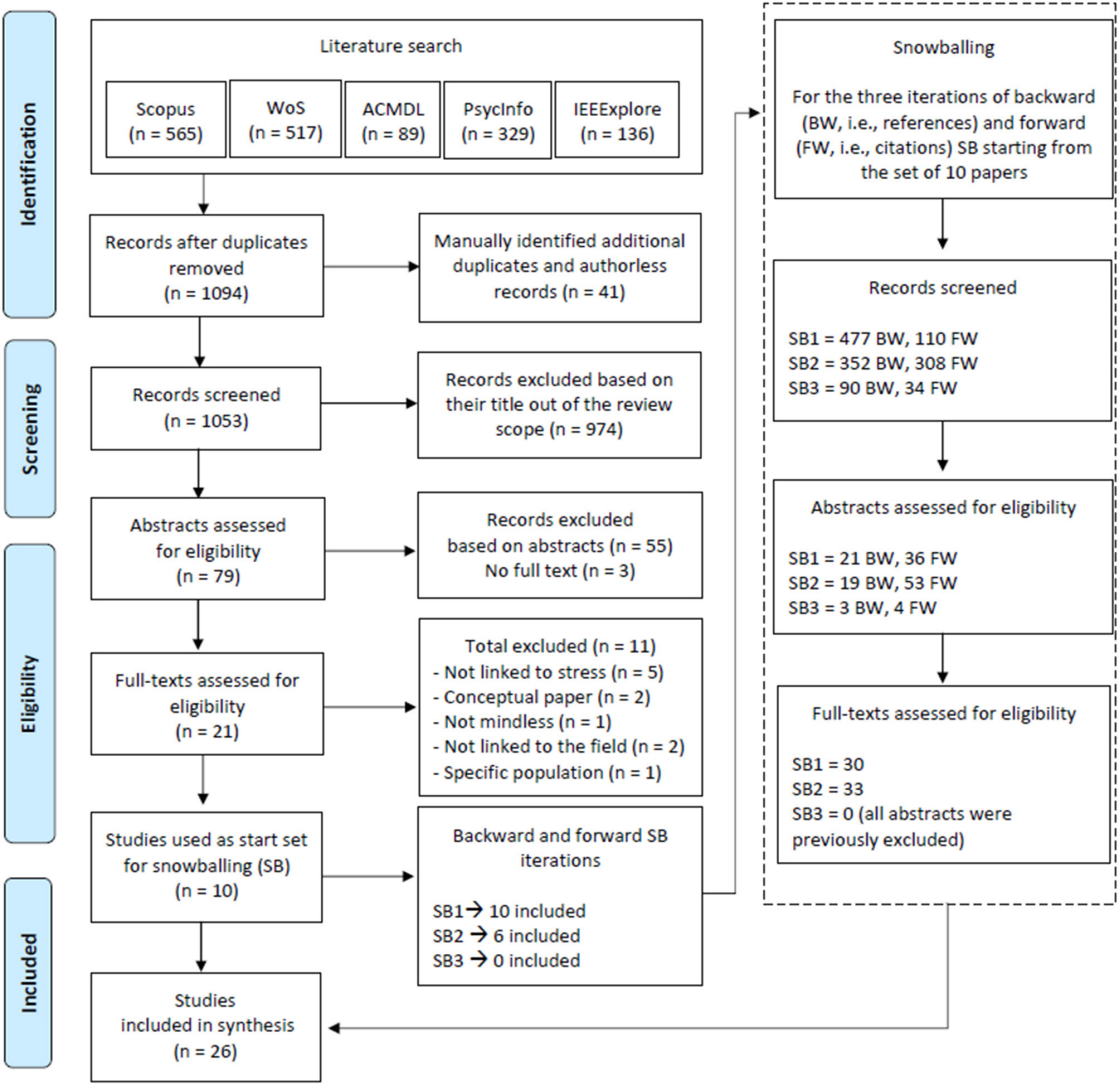
Adapted preferred reporting items for systematic reviews and meta-analyses (PRISMA) flowchart for study selection.

The exclusion process applied strict exclusion criteria throughout three stages: first, we excluded publications based on their titles, then based on their abstracts, and finally based on full texts, when available. Two independent reviewers conducted each step and solved conflicts for the first two steps. For the final selection based on full texts, a third independent reviewer resolved remaining conflicts. The full text of patents was translated when necessary using the tool provided by Espacenet “patent translate.” Due to the high variability in the terminology and the diversity of keywords used in the search, a large number of false-positive studies appeared during the review process (0.95% of initial results were included in the first set of papers). We excluded duplicates and authorless publications. Figures 3, 4 detail the PRISMA flowchart of the selection process for the two types of documents (patents and studies) included in the review.

#### Additional Sources and Selection

To complement the search for scientific publications, additional studies were identified through a backward and forward snowballing approach (Wohlin, 2014) detailed in Figure 4.

### Data Collection Process

We classified relevant information from selected full texts in two spreadsheets. The spreadsheets were divided according to publication type: patents that propose techniques directly applicable to a driving context (*actual patent*, Table 2) and studies that investigate stress regulation technologies in a driving context (*actual papers*, Table 3) or a fundamental context (*potential papers*, Table 3).

**Table 2:** Patents classification according to each regulation strategy.

**Regulation strategies**	**Patent**	**Applicant (year)**	**Driver states to regulate**	**Main goal**	**State detection technology**	**Implemented technique**
AD	CN110135355A	Univ Jilin (2019)	Two categories of emotions: positive and negative	To bring back the user at a neutral state if negative feelings are detected, using ambient displays	Recognition of facial expressions	Use of colors for ambient light arranged according to their temperature (hue) and sound arranged according to their “type” (unspecified)
AD	CN107953924A	Foshan Shenhang Tech Co. Ltd. (2019)	“Bad feelings”	To detect when the drivers awaits (e.g., traffic jams or traffic lights…) and if so, delivers him acupressure	Through the driving activity (long stops)	Haptic stimulation: steering wheel that can send acupressure
AD	KR101427926B1	Hyundai Motor Co. Ltd. Kia Motors Corp. (2019)	Stress	To detect stress and relieve it through music recommendation	Driving context & metrics (weather, speed, acceleration)	Music recommendation based on automated extraction of emotional feature of music in a database. When the stress is due to the weather, music suitable for the weather atmosphere is recommended. When the stress is caused by tension, calm music (such as classical music) is recommended.
AD	CN105873281A	China Auto Parts (Suzhou) Ind Park Dev Co. Ltd. (2016)	Moods in terms of arousal: excited and calm	To detect driver's mood and modify it using ambient displays	Not precisely specified: “mood monitoring system”	Ambient lights that can adjust their temperature to the mood of the driver
AD	CN108628205A	Changzhou Xingyu Automotive Lighting Systems Co. Ltd. (2018)	Irritability	To detect driver's mood and modify it using ambient displays	Recognition of facial expressions, voice, or input of the driver	Display of a ambient light in a specific color pre-configured by the user. Use of odors and music is also evoked
AD	CN108109228A	Guangxi Nanning Zhicui Sci Tech Consultation Co. Ltd. (2018)	Anxiety	To determine a level of anxiety using the strength of the closes of the car door. Above a certain threshold, an odor is emitted	Door closing way	Emission of odors when the door is locked too strongly
AD (audio & ambient light) CC (prompts)	CN110393540A	Zhejiang Hongquan Electronic Tech Co. Ltd. (2019)	“Unstable emotions”	To detect driver emotional instability and regulate using ambient displays or prompt him to drive safely	HR, BR (via a radar), driving characteristics	Ambient light and music. Prompts can be emitted through lights on the dashboard
AD (music & lights) CC (prompts)	CN110403617A	Guangzhou Automobile Group Co. (2019)	Mental stress	To monitor a driver's mental state and perform emotional relaxation adjustment when the driver is in a state of mental stress through ambient displays and prompts	EDA	Music recommendation, light control, voice alarm, screen display, seat vibration or steering wheel vibration
AD (music) RM (ambient air)	KR20140080727A	Korea Electronics Telecomm (2014)	Stress, excitement, boredom	To detect and recognize emotions. If negative emotion detected, to regulate them using ambient displays	Recognition of facial expression, voice, gesture, HR, HRV	Music, lighting, opening of a window, air conditioner
RM	KR101601957B1	Hong Yong Pyo (2019)	Stress, anger, drowsiness	To detect stress, anger, drowsiness through driver's voice or vehicle data, and regulate brain waves	–	Monaural beats pre-recorded, that can be modified (envelope, frequency, pitch, waveform), emitted through speakers
RM	JP2019194046A	Ecohigashinihon Corp. Kenmei Co. Ltd. Legiotec Co. Ltd. (2019)	Mental stress, tension, fatigue	To improve the comfort of a driver and other occupants who are riding in a vehicle by using hydrogen gas, or both hydrogen and oxygen gases to relax them	–	Regulation of the levels of hydrogen and oxygen in the vehicle
SM	JP2016052881A	Denso Corp. (2016)	Stress	To detect when a driver is feeling anxious and adapt the takeover request and the driving style of an autonomous vehicle	EEG, HR, BVP, EDA, driving context	Modification of vehicle behavior in autonomous mode or adjust the takeover request (time to take over or modality)
SM	JP2018020683A	Toyota Motor Corp. (2018)	Anxiety	To reduce potential anxiety due to tunnel running where light is limited	Contextual detection of side walls	Enlighten side walls of the tunnel
SM	RO127332A2	Univ Lucian Blaga Din Sibiu (2012)	Stress, discomfort	To prevent the dazzlement from light of vehicle coming from the opposite direction during nighttime that could generate stress or discomfort	–	Polarization filters to limit the amount of light reaching the eye of the driver
SM (tire noise attenuation) RM (monaural beats)	KR101085081B1	Kumho Tire Co. Inc. (2011)	Stress due to the noise of the tires	To reduce the noise emitted by the tires in order to reduce it and prevent stress of the driver due to the noise, and to obtain a frequency close to alpha range of brain waves	–	A tire design that generates human brain waves by using the monaural beat phenomenon. The offset frequency is formed in the alpha wave band (8–14 Hz) to use the noise during driving to relieve the driver's stress and improve concentration
SM (vehicle control) AD (music) CC (vocal prompt)	CN109572705A	Wuhan Luogefu Hydrogen Energy Automobile Co. Ltd. (2019)	“Abnormal driver's emotions” including distress	To detect emotional state changes (anger mode, low mode, dangerous mode) and regulate through ambient displays or prompt the driver to have a stable emotional state and a safe driving	Eye activity, interactions with passengers, posture, body temperature, speech, driving metrics (speed, acceleration, deceleration)	Depending on the emotion detected: vocal prompt, music, deceleration toward a pre-registered value or until the stop of the vehicle
SM (vehicle control) AD (music)	CN209186732U	Univ Anyang Normal (2019)	Excitement, sadness, pain and other negative emotions	To detect abnormal emotion, and recommend adapted music or modify vehicle control	HR, BR, body temperature	Music recommendation based on labeled data and speed control
SM (vehicle control) AD (music) RM (respiratory exercises)	FR2998159A1	Peugeot Citroen Automobiles Sa (2012)	Stress	To detect stress and relieve it through respiratory exercises, use of ADAS, or music recommendation	HR, BR, EDA, body temperature, voice, driving metrics	Visual or auditive respiratory exercises, use of ADAS, music recommendation pre-defined by the user
SS	KR20190103521A	Hyundai Motor Co. Ltd Kia Motors Corp (2019)	Stress	To detect the emotional state and match the GPS guidance to select an optimal path	Driving metrics or user input	GPS which provides an optimal route (previously stored) according to the state of the driver
SS (GPS recommendation) SM (vehicle control) AD (music) CC (vocal prompt)	CN102874259A	Geely Automobile Res Inst Zj Hangzhou Branch Zhejiang Geely Automobile Res Inst Co. Ltd Zhejiang Geely Holoding Group (2019)	Anger, excitement, which could include distress	To detect negative emotions, control the vehicle and regulate drivers' emotions using vehicle control, music, or vocal prompts	Recognition of facial expressions, HR, driving metrics to determine speed change and steering wheel angle	Vocal prompt or music. If driving control is detected as dangerous, limitation of the vehicle speed and increased steering wheel control, prompt of GPS notification about places to rest
SS (GPS recommendation)	JP2011027441A	Alpine Electronics Inc Honda Motor Co. Ltd MBAKK (2011)	Stress, irritability, fatigue	To search for a travel route to reduce driver stress when following a slow vehicle	Driving context	GPS recommendation if a plurality of preceding vehicles is detected.

*AD, attentional deployment; CC, cognitive change; RM, response modulation; SM, situation modification; SS, situation selection; HR, heart rate; BVP, blood volume pressure; HRV, heart rate variability; BR, breathing rate; EDA, electrodermal answer; EEG, electroencephalogram*.

**Table 3:** Studies classification according to each regulation strategy.

**Driving**	**Reg**.	**Authors (year)**	**Title**	**Stress induction**	**Implemented technique**	**Experimental conditions**	**Participants N (age)**	**Physiology measures**	**Subjective measures**	**Main outcomes**
No	AD	Amores and Maes (2017)	Essence: olfactory interfaces for unconscious influence of mood and cognitive performance	–	Odors emitted by a necklace: tea tree, peppermint, rose	Use the necklace during 3 days.	4 (mean = 29) Not naive	–	Comfort, usability, distraction, pleasantness, satisfaction, relaxation, debrief	Participants described the technology as seamless, effortless and relaxing.
No	AD	Ansems et al. (2011)	Smart photo frame for arousal feedback	Video game (snake)	Enlighted photo frame	Participants had to play to a snake game while having a small light ball that displayed colors.	Unspecified	–	Debrief	The users did not feel more stress when seeing a certain color. Colors did not influence the emotions of the users.
No	AD	Daher et al. (2020)	Reduce stress through empathic machine to improve HCI	TSST	Ambient blue light	2 conditions: TSST with light and TSST without light. Participants followed all conditions (counterbalanced).	17 (21–63)	HR HRV	Stress scale	Increase of HRV with blue light, significant reduction for no-light condition when compared to baseline. Participants under blue light reported lower stress.
No	AD (music) RM (biofeedback)	Williams et al. (2015)	Swarm: an actuated wearable for mediating affect	–	Multimodal stimulation (heat, vibration, music) delivered by a scarf according to emotional state	Brief on each module of the scarf, then participants could wear the scarf and evaluate each module	9 (18–61) Not naive	–	Semi structured interview, Usability	Interest of the participants to have a biofeedback device. Participants with disabilities rated the scarf as more useful.
No	RM	Azevedo et al. (2017)	The calming effect of a new wearable device during the anticipation of public speech	TSST	Haptic false biofeedback: wrist worn	2 groups: one with wristband OFF; one with wristband ON, vibrating with a frequency 20% lower than the rhythm measured at rest. Participant are naïve on the purpose of the study.	52 (mean = 26.4) Naive	HR EDA	STAI Ya	Lower EDA for slow HR group during the task. Lower anxiety for slow HR group after speech preparation. Task was also rated as less stressful.
No	RM	Ban et al. (2018)	Relaxushion: controlling the rhythm of breathing for relaxation by overwriting somatic sensation	–	Haptic stimulation: cushion that can adjust its size	2 types of breathing tempo: 7 resp/min or 15 resp/min. Participants are naive of the purpose of the study.	5 (unknown) Naive	BR	Debrief	Participants BR adjusted to the motion of the device. Low level of distractions.
No	RM	Bergstrom et al. (2014)	Using music as a signal for biofeedback	–	Music tempo and volume modulation according to heart rate	3 conditions: listening to pre-recorded music; Sonification biofeedback; Musical biofeedback. Participants are instructed to increase or decrease their arousal level in each condition.	24 (mean 28.2) Not naive	HR BR	Bodily Awareness Questionnaire, Debrief	Facilitating effect of musical biofeedback to modulate arousal through BR, when compared to sonification or music alone.
No	RM	Choi and Ishii (2020)	Ambienbeat: wrist-worn mobile tactile biofeedback for heart rate rhythmic regulation	Physical exercise	Haptic biofeedback: wrist worn	3 tasks: sit still; Sit still after physical exercise; draw with a mouse. Participants had a tactile, visual or auditive stimulation at 60 or 120 beats per minute (bpm).	12 (18–60)	HR HRV	Modality preference	Better effects of tactile stim to reduce stress level: increased HRV and fastest HR decrease after jumping with the tactile stimulation. Higher preference to tactile and lower disturbance level.
No	RM	Costa et al. (2016)	Emotioncheck: leveraging bodily signals and false feedback to regulate our emotions	TSST	Haptic biofeedback: wrist worn	2 groups: one with wristband ON, one with wristband OFF. For the group ON, 3 conditions: vibrations at 60 bpm with naïve participants (vibration group); Vibrations at 60 bpm with participants informed that the vibrations represented HR (slow HR group); Vibrations at real HR (real HR group).	67 (19–30) Naïve/not naive depending on the condition	HR	STAI Ya, STAI Yb, Distraction	Lower anxiety scores for slow HR group. The intervention was rated as not distractive.
No	RM	Costa et al. (2019)	Boostmeup: improving cognitive performance in the moment by unobtrusively regulating emotions with a smartwatch	Mental calculation	Haptic biofeedback: Applewatch	2 groups: one with slow biofeedback (slow HR); one with increased biofeedback (fast HR).	72 (18–25) Not naive	HR HRV	STAI Ya, Distraction	HRV increased for slow HR group. HR decreased for fast HR group. Performance increased for slow HR group. Increased reaction times for slow HR. The technique was not distractive for the participants. Fast HR increased anxiety. Slow HR reduced anxiety.
No	RM	Cuijpers et al. (2019)	Psychophysiological stress control via heart rate entrainment	TSST	Heartbeat-like sound	3 groups: one with slow biofeedback; one with real biofeedback; one with no biofeedback.	30 (unknown)	HR EDA	STAI Ya	No significant results.
No	RM	Fedotchev et al. (2017)	Effects of musical acoustic signals controlled by the subject's eeg oscillators	–	Sound modulated according to EEG oscillations	2 types of sound: one that varies according to EEG, another presenting the same variations together with a 1-Hz rhythm.	17 (23–55)	HR HRV EEG	WAM scale (well-being, activity, mood)	Increase of alpha and beta waves for the condition with the 1-Hz rhythm. Significant increase in well-being and mood.
No	RM	Hamon et al. (2018)	Exploring biofeedback with a tangible interface designed for relaxation	N-back task	Visual biofeedback: ambient light from a flower	4 groups: focused attention required while the light varied according to HR and BR (dynamic light); focused-static light (the light vary at a fixed rhythm of 6 bpm); ambient	36 (mean = 23.8)	HR	STAI Ya USE survey (usability)	No significant results.
						(no focused attention required)-static light; ambient-dynamic light.	
No	RM	Harris et al. (2014)	Sonic respiration: controlling respiration rate through auditory biofeedback	–	Music modulation according to breathing rate	2 types of music manipulation: audio tract layering (manipulation of the quality of audio or noise addition).	6 (20–59) Not naive	BR	Survey on attitude toward the technique	Lower BR with audio modification. Participants preferred noise addition over track layering.
No	RM	Kim et al. (2018)	Affective and autonomic response to dynamic rhythmic entrainment: mechanisms of a specific music therapy factor	Stroop task, Quesions, Mental calculation	Music tempo modulation according to heart rate	2 groups listened to music: one group with adaptative tempo that follow participant's HR then gradually decrease; One group with fixed tempo at 70 bpm. Both groups had control condition with no music.	30 (mean = 26.9)	HR BVP	Stress scale, Well-being scale	Adaptative tempo group: Strongest increase in the peripheral blood flow (indicating better stress recovery). Strongest increase in global well-being. Slight stress reduction (not significant).
No	RM	Leslie et al. (2019)	Engineering music to slow breathing and invite relaxed physiology	Oddball task	Music recommendation according to breathing rate	4 conditions: silent; fixed tempo; personalized tempo; personalized envelope.	19 (19–55) Naive	HR HRV BR BRV	–	Decrease in BR across the conditions (baseline > fixed tempo > personalized envelope > personalized tempo). BRV increased when compared with baseline. EDA reduction for personalized tempo. Increase in z-scored interbeat intervals for the condition fixed tempo when compared to baseline.
No	RM	Lopes and Campos (2019)	SCAARF: a subtle conditioning approach for anxiety relief facilitation	Various daily stressors	Haptic biofeedback and sound conditioning: scarf	1 group of participant has to use the scarf at least 10 min/day during 3 weeks. During the first phase, when the user gets stressed, an app can guide him through respiratory exercise, which is conditioned with a sound. During the second phase, the participant only has the sound.	7 (mean = 31)		Debrief	Technology rated as subtle (scarf and sound). Less stress was felt by participants for the conditioning phase.
No	RM	Sato and Moriya (2019)	Respiration rate change induced by controlling the phasic relationship between melodic sound and respiration	–	Music modulation according to breathing rate	6 types of target phases, defined by their shift with breathing patterns, 3 melodic sounds (pop, rock, classic).	10 (20–50) Naive	HR BR BRV	–	Significant differences in BR depending on the phase selected. Higher BR when the target phase is different from participant breathing pattern.
No	RM	Yu et al. (2018)	Delight*:* biofeedback through ambient light for stress intervention and relaxation assistance	Mental calculation + time pressure	Visual biofeedback: environment light modulation according to HRV	Three groups: control without biofeedback, biofeedback with warm light, biofeedback with cold light.	12 (25–35) Not naive	HR HRV	Stress scale, relaxation scale, distraction	Lower change of HR when compared to control condition. Higher change in HRV for warm BF compared to both control and cold BF. Stress increase more for the control condition than for both BF. Stress increase more for warm than for cold BF. Participants prefer cold BF and find it more relaxing and less distracting
No	RM	Zhou et al. (2020)	The calming effect of heartbeat vibration	–	Haptic biofeedback: vibrations according to heart rate	Participants had haptic biofeedback during 3 sessions composed of resting phases and stimulation phases.	21 (mean = 35.7)	HR HRV	STAI Ya	HRV increased and HR decreased compared to the two rest conditions, in all three sessions. There is a main effect of the sessions, translating into an increase in RMSSD and decrease in HR.
Yes	AD	Hu et al. (2015)	Safedj: a crowd-cloud codesign approach to situation-aware music delivery for drivers	–	Music recommendation according to emotional state	4 groups: 1 without music; 1 with music recommendation by default player; 1 with music recommendation according to user's mood.	48 (unknown)	HR HRV	–	Increased reduction of the fatigue and of the negative moods experienced when the system recommended music.
Yes	RM	Balters et al. (2020)	Calm commute: guided slow breathing for daily stress management in drivers	TSST Heavy metal song	Respiratory guidance through haptics in the seat	2 groups: 1 with haptic guidance; 1 without (control). Counterbalanced order of stress induction methods.	24 (mean = 40.2) Not naive	HR HRV BR	Stress scale, Physical tension, Affect grid (arousal and valence)	Normal driving: decrease of 15% of BR (no significant). Post stress driving: reduction of 25.3% of BR, increase in HRV. Participants expressed to have calming effect and low distraction from the guidance.
Yes	RM	Bhandari et al. (2015)	Music-based respiratory biofeedback in visually-demanding tasks	Video game (racing)	Music modulation by adding of a white noise according to breathing rhythm	4 conditions: no music no feedback (control), music only, auditory biofeedback (only white noise reflecting BR), musical biofeedback (music with white noise).	28 (22–35) Not naive	HR HRV BR EDA	Valence, Calmness	Lower arousal (BR & EDA decrease and higher HRV) for musical biofeedback. Lower EDA for musical biofeedback compared to auditory biofeedback.
Yes	RM	MacLean et al. (2013)	Moodwings: a wearable biofeedback device for real-time stress intervention	Driving context	Visual biofeedback: moving wings of a butterfly on the wrist according to arousal	6 conditions: 2 modalities for butterfly (actuated, stationary) × 3 driving actions (easy, maneuvering, unexpected event). Participants are informed of the purpose of the technology.	11 (unknown)	EDA	Stress scale	Driving performance was better in the actuated condition (driving more safely). Users felt more stress when mood swings was in actuator mode.
Yes	RM	Paredes et al. (2018b)	Just breathe: in-car interventions for guided slow breathing	Driving context	Respiratory guidance through haptics in the seat or voice guidance	2 groups: Manual mode and autonomous mode. Two guidance modalities: voice or haptics, both with a frequency lowered by 30% according to breathing baseline. Two driving scenarios: one stressful urban driving, one highway driving.	24 (18–64)	HR HRV BR EDA	Stress scale, Distraction	No significant differences for EDA. Decrease in BR & increase in HRV with both audio and haptic guidance. No difference in driving performance. Stress reduction trend. Haptic is rated as subtle but participants noted they would not focus on the device if the situation is too stressful. Participant preferred haptic modality.
Yes	RM	Zepf et al. (2020)	Empathicgps: exploring the role of voice tonality in navigation systems during simulated driving	–	GPS voice modulation according to emotional state estimated via EDA	3 voice modalities: congruent (biofeedback adapted to the arousal level); incongruent (BF unadapted to the arousal level); neutral (constant calm voice).	18 (22–58) Not naive		Stress scale, affect grid (arousal and valence), pleasantness of the voice	Brake intensity increased in the incongruent condition. No differences between congruent and neutral. Incongruent voice is associated with lower arousal and higher stress. Congruent voice is associated with higher pleasantness.

*AD, attentional deployment; CC, cognitive change; RM, response modulation; SM, situation modification; SS, situation selection; HR, heart rate; BVP, blood volume pressure; HRV, heart rate variability; BR, breathing rate; BRV, breathing rate variability; EDA, electrodermal answer; EEG, electroencephalogram*.

In the patent spreadsheet, we classified information according to the following variables: (1) regulation strategy identified, (2) patent number, (3) applicant of the patent and year of publication, (4) type of emotion the patent focuses on, (5) main goal of the patent, (6) technique employed for emotion detection if applicable, and (7) regulation technique implemented.

In the study spreadsheet, we classified information according to the variables listed below: (1) regulation strategy identified; (2) authors and year of the study; (3) title of the study; (4) presence of stress induction and, if so, the task used; (5) main technique evaluated; (6) experimental conditions; (7) characteristics of the sample, including sample size, average age, or range; (8) metrics to evaluate the efficiency of the technique employed including physiological and subjective measurements; and (9) main outcomes of the technique presented.

No quality assessment, in terms of statistical power, of the contributions was performed.

### Classification Process of Regulation Strategies Found

A classification chart (Figure 2) was designed based on the explanations provided by Gross (2014), and each included record went through this chart to determine at what stage it regulates stress is effectively. It was first considered that some techniques can be linked to several regulation strategies: for instance, a technique that uses music to regulate stress could be classified as situation modification (as the global context will be changed by adding the music), but music will only be efficient to regulate once the stage of attentional deployment is reached. In order to draw clear boundaries, we have chosen to include in each strategy the techniques that are effective only once the stage of regulation where the technique is at its maximal efficiency has been reached. However, selected patents often propose several techniques based on several regulation strategies.

For instance, using this chart, a study proposing a regulation strategy relying on biofeedback technology could be classified this way:

The technology does not allow avoiding the situation, directly modifying the stressor, nor changing the perception and subsequent evaluation of the situation but is indeed based on a change of the physiological stress response.

## Results

### Patent Selection

The search performed on 03/07/2020 on EspaceNet provided a total of 490 unique records. Of these, 364 were excluded based on their title, mostly because they were not linked to the review field. Among the remaining 126 patents, 53 additional records were excluded after reviewing the abstract. Of the remaining 73 patents, 52 additional records were excluded based on their full description.

Main reasons for full-text exclusion were the following: not being linked to stress regulation (*n* = 21), not being related to the field (*n* = 13), not being mindless (*n* = 7), no full text in English found (*n* = 5), and being on detection only (*n* = 6).

The Cohen's kappa score for agreement between the two reviewers for the full text screening phase was κ = 0.66. According to Landis and Koch (1977), this is a strong agreement value.

Finally, a total of 21 patents were identified for inclusion in the review (for the full patent list, see the **Appendix**).

### Characteristics of Included Patents

It was not possible to determine from the patents whether participants were involved to test the techniques proposed.

**Figure 5:**
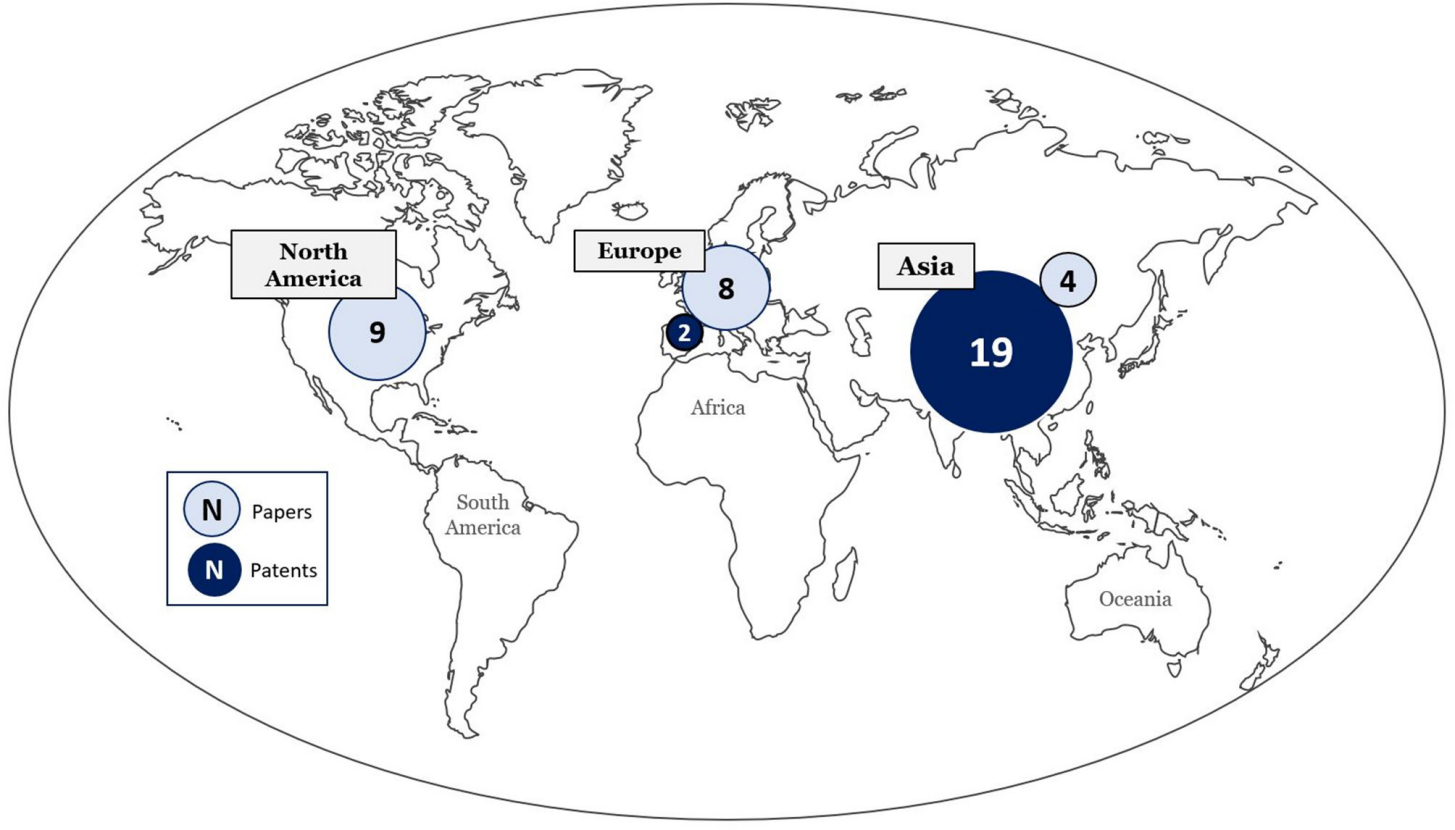
Continents of origin for patents and studies, international collaborations (*n* = 5) are not displayed.

Patents retrieved were mostly from Asia, as shown in Figure 5. Countries of origin are China (*n* = 10), Korea (*n* = 5), Japan (*n* = 4), Romania (*n* = 1), and France (*n* = 1).

Several modalities are employed for regulation. They include audio display such as music or sounds (*n* = 2), visual display such as lights (*n* = 5), haptics such as acupressure (*n* = 1), odors (*n* = 1), or others modalities such as vehicle control or ambient air changes (*n* = 2). Several patents employ a combination of modalities, such as audio, visual, and other (*n* = 2); audio, haptic, and visual (*n* = 1); visual, audio, and odors (*n* = 1); audio and other (*n* = 3); audio and haptic (*n* = 1); and audio and visual (*n* = 2).

Therefore, strategies identified in patents were situation selection (*n* = 2), situation modification (*n* = 3), attentional deployment (*n* = 6), and response modulation (*n* = 2). None of the patents are only using a cognitive change-based regulation strategy. Several patents propose at least two regulation strategies, including situation modification and attentional deployment (*n* = 1); attentional deployment and response modulation (*n* = 1); attentional deployment and cognitive change (*n* = 2); situation modification and response modulation (*n* = 1); situation modification, attentional deployment, and cognitive change (*n* = 1); situation modification, attentional deployment, and response modulation (*n* = 1); and situation selection, situation modification, attentional deployment, and cognitive change (*n* = 1).

**Figure 6:**
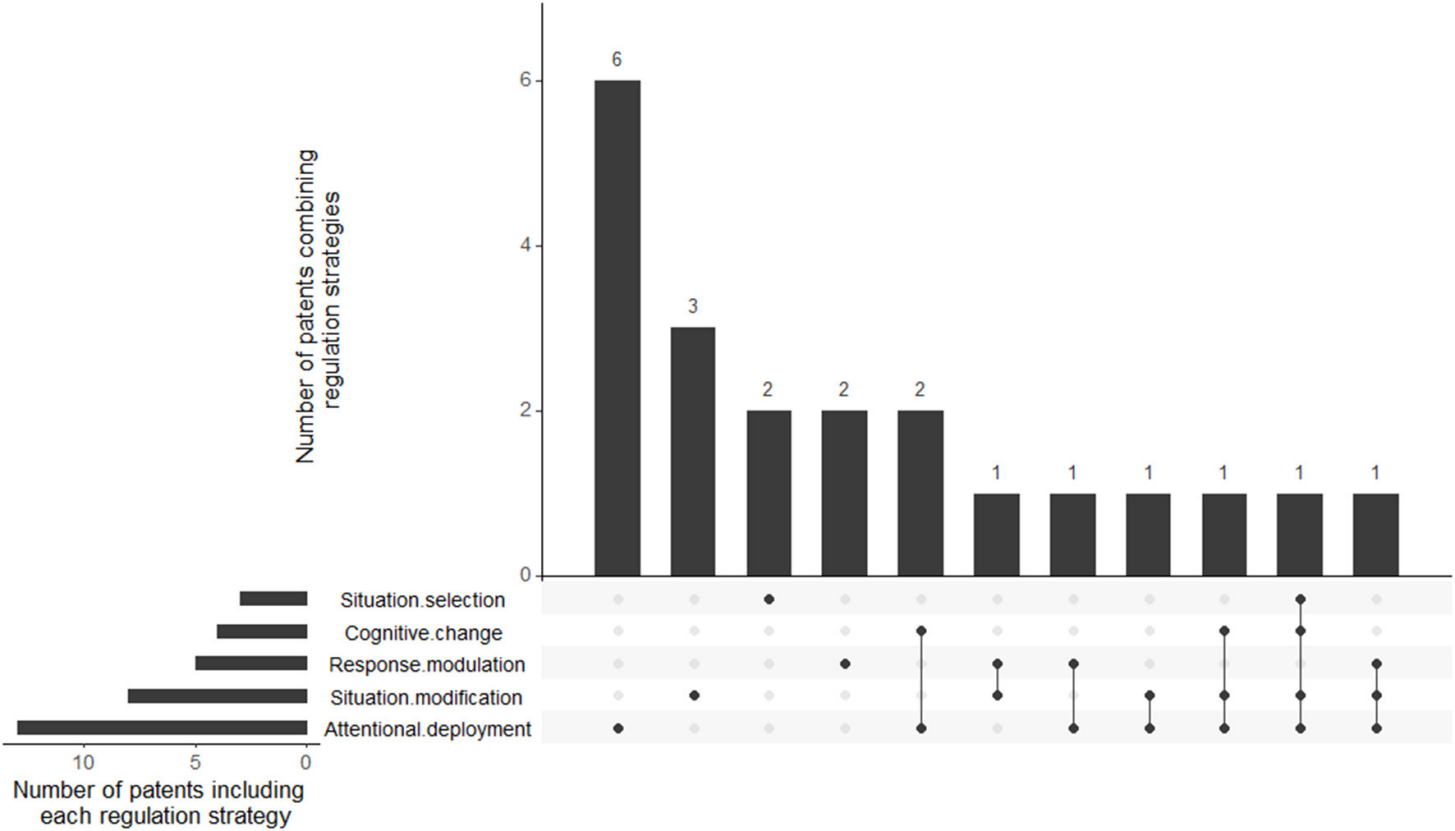
Upset plot displaying the number of patents for each strategy's combination.

Figure 6 is an upset plot (Lex et al., 2014) displaying the matrix layout of the combination of regulation strategies in the patents. Each row of the matrix represents a set, and each column displays the intersection between sets of regulation strategies when they exist (lines between the dot). The histogram above the matrix shows the number of patents for each intersection, while the histogram next to the matrix exposes the number of patents that include each regulation strategy.

All of the patents but four (see Table 2) included the possibility of measuring the state of the driver (through various biometrics) or the driving metrics and context.

### Study Selection

The combined search in Scopus, Web of Science, ACMDL, PsycInfo, and IEEExplore, performed on 03/07/2020, provided a total of 1,053 unique records. Of these, 974 were excluded because the topic studied was not relevant for the review topic (e.g., Optics or Engineering). Among the remaining 79 documents, 55 additional records were excluded after reviewing the abstract due to being unrelated to stress, or not being mindless; 3 more records were excluded because the full text could not be found. Of the remaining 21 articles, 11 were excluded based on the full paper.

The reasons for exclusion were the following: not being linked to stress regulation (*n* = 5), being a conceptual paper or no concrete technique tested (*n* = 2), not being linked to the field of the review (*n* = 2), using an invasive technique such as electrostimulation (*n* = 1), not being mindless (*n* = 1), and application on a specific population (children, *n* = 1). One excluded article met more than one exclusion criteria.

The Cohen's kappa score for agreement between the two reviewers for the full text screening phase was κ = 0.62. According to Landis and Koch (1977), this is a strong agreement value.

Due to the low number of included studies, the 10 remaining studies were used as a starter set to apply a backward and forward snowballing procedure, according to guidelines provided by Wholin (2014). Backward snowballing refers to review the references contained in the start set papers, while forward snowballing refers to review records citing the start set papers. Each iteration uses the included studies from the previous iteration as start set. The detail of the three iterations is provided in Figure 4. At the end of the snowballing process, 16 additional papers were included in the review, bringing the total number of included studies to 26. These additional studies met the same criteria as the studies from the initial set.

### Characteristics of Included Studies

Included studies originated from USA (*n* = 9), Japan (*n* = 3), Netherlands (*n* = 2), United Kingdom (*n* = 1), Germany (*n* = 1), Switzerland (*n* = 1), France (*n* = 1), Spain (*n* = 1), Portugal (*n* = 1), Russia (*n* = 1), as displayed in Figure 5.

Five additional papers are international collaborations from: Netherlands and Italy; Canada, China, France, and Sweden; USA and Qatar; USA, Norway, and Germany; and USA and Germany.

Papers could be classified as *actual papers* (*n* = 6) when the technology was tested in a driving environment; otherwise, they were classified as *potential papers* (*n* = 20).

The cumulative number of participants in the studies is about 603, including 226 men—the exact number of participants cannot be known since one study does not provide this information. Similarly, five studies do not indicate the number of men involved among the participants.

The population type is mostly composed of young adults that are university students (*n* = 6 studies), university employees (*n* = 3 studies), general population (*n* = 3 studies), drivers in the studies that are conducted in a driving context (*n* = 6 studies), participants that are subject to anxiety (*n* = 1 study), or participants with disabilities (*n* = 1 study) including impaired vision, impaired audition, and autism.

Several modalities are employed in studies to display the intervention on the participants. Modalities include audio display (*n* = 10), visual display (*n* = 5), haptics (*n* = 8), and odors (*n* = 1). Some studies employ a combination of modalities, such as haptic and visual (*n* = 1) and haptic and sound (*n* = 1).

**Figure 7:**
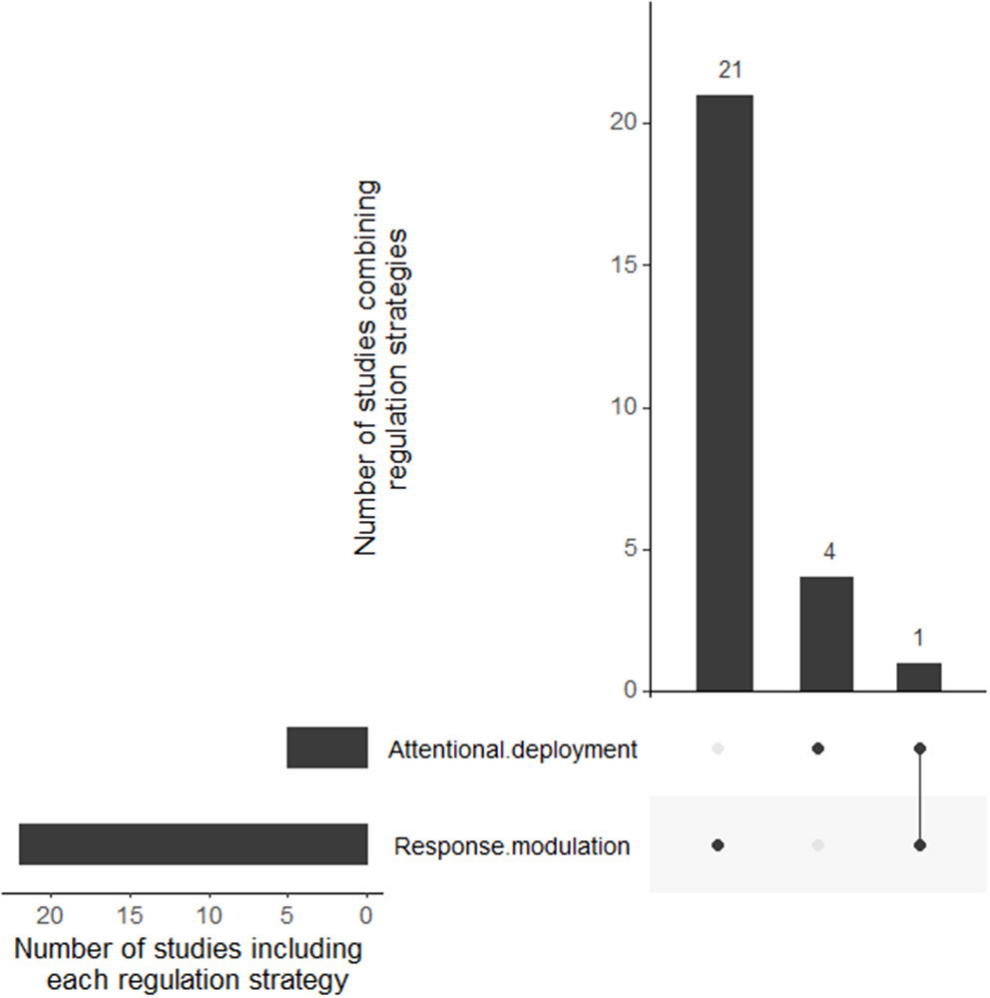
Upset plot displaying the number of studies for each strategy's combination.

We employed the same classification chart used for patents to determine the regulation strategy employed by studies (Figure 2). Strategies identified in studies are the following: attentional deployment (*n* = 4) and response modulation (*n* = 21). None of the studies are using situation selection, situation modification, or cognitive change. One study tested two regulation strategies, including attentional deployment and response modulation. Figure 7 represents the number of studies for each regulation strategy and overlaps.

**Figure 8:**
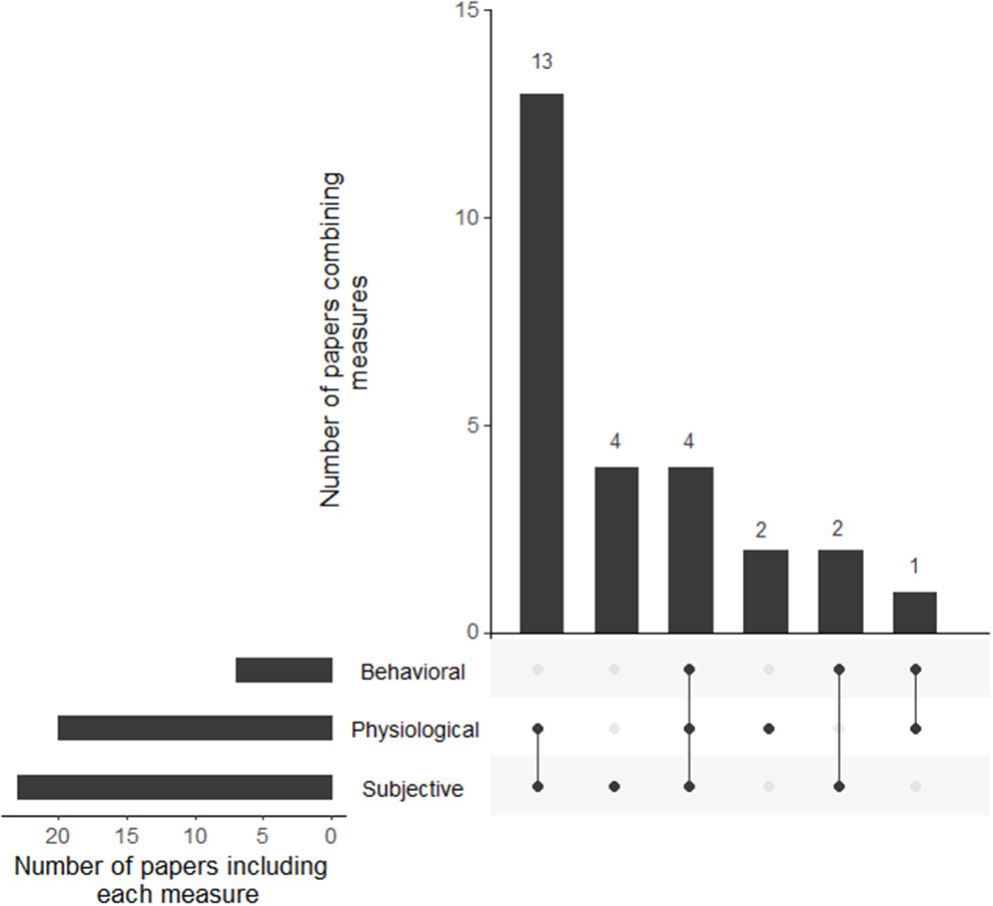
Upset plot displaying the number of studies for each measurement's combination.

Data collected in the studies include physiological metrics (*n* = 20), behavioral metrics (*n* = 7), and subjective metrics (*n* = 23). Details of this distribution can be found in Table 3. Figure 8 gives an overview of overlaps for all papers.

Physiological metrics employed are mostly linked to cardiac activity (*n* = 18), respiratory activity (*n* = 8), and electrodermal activity (*n* = 4). One study employs electroencephalography together with cardiac activity. Among the behavioral metrics, two studies employ measurement of performance in the stressful task, while five of the six driving studies (“actual papers”) employ measurement of the driving performance using metrics such as acceleration, steering wheel, and lane departure.

The subjective measures are mostly based on the stress scale (*n* = 7), State–Trait Anxiety Inventory (STAI-Y, *n* = 6), interview and postexperimentation debrief (*n* = 6), or other questionnaires.

## Discussion

The Gross emotion regulation model (2014) reveals itself as a pertinent reading grid to classify the selected publications. Therefore, the discussion has been divided into five sections linked to the different Gross emotion regulation stages. Of note, most records do not explicitly provide the regulation stage involved, so a classification was applied as described in *Methods*. In addition, some publications can fit in more than one section as they evaluate several technologies (see Figures 6, 7). Each section will introduce the corresponding stage; then, the discussion will explain the methods found (Tables 2, 3) involving this stage for: *actual patents*, thus giving an idea of applications in the automotive industry; *actual papers* reporting strategies in a driving situation (naturalistic or simulated); and *potential papers* consisting in scientific contributions tested outside the driving context but which could be suitable due to their intrinsic mindless characteristics and their feasibility in the car.

### Situation Selection

This strategy is the first possible stage to regulate stress. It refers to the avoidance of a stressful situation by choosing an alternative situation less likely to elicit stress. Therefore, acting on this component is tantamount to anticipating and avoiding stressors. The application of the strategy is here specific to the driving context.

#### Actual Patents

The main solution consists in selecting a road that is likely to be less stressful. CN Patent No. 102874259A (2013) proposes to use the GPS to suggest the closest locations to rest. Likewise, JP Patent No. 2011027441A (2011) uses context recognition to determine if there is a lot of traffic surrounding the user's car. If so, the GPS picks a road with less traffic. Similarly, KR Patent No. 20190103521A (2019) provides an optimal road according to the automatically detected state of the driver or when the driver triggers the system. Indeed, the road can be parametrized in advance by the driver himself. This is in line with Gross's (2015) observation that it would be hard to anticipate “how one will feel in different situations.” Indeed, this suggests that personalization is necessary according to each user's predisposition to feel distressed in a particular situation. Therefore, to allow the user to (1) select in advance a potentially relaxing road and (2) to trigger the guidance seems particularly pertinent.

#### Situation Selection: General Discussion

Current apps such as Google Maps (which may provide the road with least traffic) can share the same approach to the patents presented below. Nonetheless, an essential difference exists: the device is aware of the state of the user or the context directly surrounding the car and the possible repercussions on drivers' well-being. Among the six *actual papers* that have tested stress regulation within the driving context, none of them were identified as exploiting the situation selection strategy. Studies mainly focus on stress response given a particular situation but do not propose to “avoid a situation.” Indeed, while driving, it is difficult to find other solutions that fall within situation selection (SS) other than choosing the road with least traffic since other stress sources like weather are not possible to avoid.

### Situation Modification

Situation modification refers to altering the stressful situation, without avoiding it. Included in this section are contributions that proposed to act either on the stressful situations or on the stressors. This means that techniques implying a global modification of the context features not related to stressors (e.g., the techniques modifying the cockpit environment by an ambient display) were not included.

#### Actual Patents

Several patents propose to modify the driving context via the modification of the vehicle's controls. Indeed, CN Patent No. 102874259A (2013), CN Patent No. 109572705A, 2019), and CN Patent No. 209186732U (2019) propose to limit the speed of the vehicle and to control the steering wheel. FR Patent No. 2998159A1 (2012) suggests connecting conventional ADAS (e.g., park assistance, speed limitation) with stress detection. In the autonomous context, JP Patent No. 2016052881A (2016) proposes to adapt the behavior of the vehicle if stress is detected by adjusting the modality of the takeover request that could eventually generate stress.

Three patents are based on very specific driving contexts. KR Patent No. 20110034326A (2011) proposes to modify the structure of the tires to attenuate the noise and to produce monaural beats (this patent will be explained in detail in the response modulation section). JP Patent No. 2018020683A (2018) presents a reduction in stress while crossing a tunnel. The patent detects the side walls and illuminates them. RO Patent No. 127332A2 (2012) proposes to attenuate the dazzlement from incoming vehicles' lights during nighttime.

#### Situation Modification: General Discussion

Given its prevalence in patents, a question that could arise is: Would the vehicle control be an efficient way to regulate stress? On the one hand, the situation can be modified by limiting or reducing speed, thus becoming less stressful. On the other hand, this countermeasure could entail more stress due to the lack of the feeling of control (Kim and Diamond, 2002). Patents proposing to rely on conventional ADAS to manage the situation when necessary can be linked to some studies suggesting that ADAS such as active park assist, active city stop, or adaptive cruise control could be an efficient way to regulate stress while driving (Chung et al., 2019 for a review).

Situation modification techniques, similarly to situation selection, seem specific to the driving context since it would be hard to interact with personal factors. None of the *potential papers* nor *actual papers* investigated situation modification, as its implementation would be very specific to the investigated context.

### Attentional Deployment

Attentional deployment refers to an attentional redirection of the user (toward either internal or external focus). The regulation is achieved by diverting the driver from stressors. This attentional redirection can be toward a neutral element or toward an element that can remind relaxing or pleasant memories. To guarantee the mindless approach, only contributions relying on peripheral attention (Bakker and Niemantsverdriet, 2016) were considered.

#### Actual Patents

CN Patent No. 107953924A (2018) proposes to use the steering wheel to send acupressure on the hands of the driver to relax him during traffic jams or long traffic lights. CN Patent No. 105873281A (2016), CN Patent No. 110393540A (2019), and CN Patent No. 108628205A (2018) propose lighting control that adjusts the temperature (i.e., the color) of the ambient lights according to the mood of the driver or to a preset value. KR Patent No. 20140080727A (2014) also proposes to modify the ambient light as well as the ambient air in the vehicle using the air conditioner and windows (further detailed in *Response Modulation*).

Including auditory modality, CN Patent No. 110403617A (2019) proposes a musical modification together with lighting control. CN Patent No. 110135355A (2019) also uses the combination of sounds and colored lights when a negative emotion is detected through facial expressions. Arguably, music is one of the preferred techniques to reduce stress given the numerous patents using it: CN Patent No. 109572705A (2019), CN Patent No. 102874259A (2013), CN Patent No. 110403617A (2019), Patent No. 209186732U (2019), KR Patent No. 101427926B1 (2014), KR Patent No. 101427926B1 (2014), and FR Patent No. 2998159A1 (2012). CN Patent No. 110393540A (2019) and CN Patent No. 108628205A (2018) present classification systems to play the driver's “favorite music” or automatically selected music according to emotional characteristics.

Breaking from the mold, CN Patent No. 108109228A (2018) proposes to generate an odor to regulate a stressed state. This odor is triggered by the force at which the driver closes the door. CN Patent No. 108628205A (2018) also mentioned odors as an interesting perspective to regulate emotions but does not focus on this technique.

#### Actual Papers

Exploring regulation in a driving context, Hu et al. (2015) uses a music recommendation system to elicit relaxation. Similarly to some patents presented above, music tracks are classified according to the emotional valence and are delivered to enhance driver's mood. Their results show a reduction in fatigue and negative moods detected through an algorithm.

#### Potential Papers

Daher et al. (2020) evaluated the efficiency of ambient blue light to reduce stress induced by a mathematical task. The participants reported lower stress levels under this light. While also using lights, Ansems et al. (2011) introduce a photo frame that can light up when distress is detected. The idea is to elicit pleasant or relaxing memories by directing the attention toward a photograph that has significant value.

Williams et al. (2015) introduce a wearable scarf that can actuate (vibration, cooling, weight) according to the user emotions.

Amores and Maes (2017) tested various odors to elicit relaxation. The odors used were tea tree, peppermint, and rose fragrances and were delivered using a necklace containing the scents that could be triggered manually (on user's request) or automatically (based on biosignals).

#### Attentional Deployment: General Discussion

The technologies presented in this section should be compatible with driving and be efficient without requiring focused attention. Therefore, the methods are mainly linked to music recommendations and ambient light. Music is well known to reduce stress and can have a beneficial impact on both psychological and physiological aspects (de Witte et al., 2020). Thus, to provide a recommendation system adapted to the driver's mood seems to be a suitable technique. Concerning the use of ambient lights, it has been shown that colors and light can have various impacts on human psychology (Elliot, 2015), although it seems unclear whether specific colors have a specific impact or if it depends on personal factors (Johnson and Toffanin, 2012).

Of note, in the case of complete autonomous vehicles, attentional deployment could present more possibilities, since attention would not have to be always centered on the driving environment (Pfleging et al., 2016). For instance, the technique based on the use of an enlightened photo frame to attract attention (Daher et al., 2020) could be taken further, with for instance multiple photographs or even videos that elicit pleasant memories. Attentional deployment technologies are often complemented with another, such as biofeedback technologies.

### Response Modulation

Response modulation is associated with altering the experiential, physiological, or behavioral stress response of the organism. Publications mainly altering the physiological component of user's response were included. This alteration can be done in a passive or active way. The former mostly relies on the deliverance of truthful biofeedback reflecting the physiological state of the user, while the latter tries to obtain a so-called “entrainment effect” to actively align the user response to a specific state (e.g., to a specific breathing or cardiac rhythm) either through guidance, through false biofeedback, or using another alteration.

#### Actual Patents

KR Patent No. 101601957B1 (2016) proposes to use monaural beats emitted through speakers. In a similar manner, KR Patent No. 20110034326A (2011) proposes to generate a monaural beat from modification of the tires' structure. Both patents try to generate a frequency close to the alpha range of brain waves to induce a relaxed state (Chaieb et al., 2017).

KR Patent No. 20140080727A (2014) proposes to modify the ambient air in the vehicle using the air conditioner and windows. In an interesting way, JP Patent No. 2019194046A (2019) suggests modulating the physiological response by increasing the oxygen and hydrogen levels within the car to induce relaxation. A more diverting technique linked to breath regulation proposes to visually prompt respiratory exercises (FR Patent No. 2998159A1, 2012).

#### Actual Papers

MacLean et al. (2013) displayed biofeedback on the wrist of the driver, through a device called “Moodwings.” This involves a butterfly that moves its wings according to the stress level, determined via electrodermal activity. This technique is original and well-designed, but the results showed that the participant felt more stressed when the system was activated, although driving performance was improved. Authors explain these results by lack of training. Following the same line, but using “musical biofeedback,” Bhandari et al. (2015) modified a music track by adding white noise varying in intensity depending on the breath rhythm of the driver. The physiological and subjective results showed that their arousal level was significantly lower.

Zepf et al. (2020) used a different auditory modality to regulate driver's emotions. In their study, the voice of the “empathic GPS” guidance system was modified to decrease the arousal of the drivers, determined from their electrodermal response. The voice congruent to the state of the driver (in the sense that if the user was stressed, the voice was calmer) was subjectively associated with higher pleasantness, but no difference for stress was found. Such “empathic GPS” can be considered as a kind of biofeedback as the voice adaptation followed EDA.

Using a haptic modality, an entrainment effect was induced by Paredes et al. (2018b) and Balters et al. (2020) who prompted breathing instructions by using haptic guidance embedded in the seat. Participants of both studies rated this method as subtle and calming, and a significant reduction in breathing rate and augmentation in HRV was observed.

#### Potential Papers

Lopes and Campos (2019) used a scarf that delivered a sound to induce relaxation. This sound was previously conditioned together with respiratory exercises to induce a relaxation state. Although this method can be linked to cognitive behavioral therapies, it presents the disadvantage of requiring a previous conditioning phase to be efficient. This method was rated as subtle, although it was more relaxing during the conditioning phase.

Kim et al. (2018) applied a modulation of music according to the participant's HR. In their study, the music first matched participants HR, and then, a reduction was applied. In the same manner, but using a periodic sound linked to HR, Cuijpers et al. (2019) tried to induce an entrainment effect to regulate the physiological response. Similarly, Bergstrom et al. (2014) modified music tempo according to HR.

Focusing on respiration, Harris et al. (2014) modified music by adding white noise that varied according to participants' BR. Leslie et al. (2019) also tested various music modifications depending on the respiratory signal. Sato and Moriya (2019) used music by controlling the synchronicity with acoustical timing (phrases, pitch change). These results suggest that the degree of synchronization of the music with the physiological response can induce effective changes in the stress response.

Fedotchev et al. (2017) proposed an alternative way to modify music following biosignals by using electroencephalogram (EEG). The EEG signal is converted into a flute-like sound that varies in terms of pitch and intensity. The experimentation tests the effect of adding 1 Hz rhythm to this signal. Even though this method could be uncomfortable to apply in a car due to the limits of EEG systems, it presents interesting results, such as a significant increase in well-being, as long as increased brain alpha and beta waves.

Williams et al. (2015) used haptic modality to deliver, by means of a scarf, biofeedback to the user in the form of vibrations. Disabled participants found it especially useful. Using also haptic modality, Costa et al. (2016) delivered false biofeedback in the form of vibrations on the participant's wrist. The authors introduced a deceiving condition where the vibration's frequency was always at 60 bpm (lower than the real HR). The participants felt lower levels of anxiety and found the technique as subtle. They replicated their study (Costa et al., 2019) including a condition with 30% reduced HR biofeedback, which induced an HRV decrease, improved performance, and reduced anxiety. A study using similar wrist-worn (Azevedo et al., 2017) found a consistent pattern of results with a reduced biofeedback, since the arousal level (measured by electrodermal activity) and self-reported anxiety were lower. However, a replication study (van der Velden and Lakens, 2020) failed to replicate this effect. Using a similar device delivering a heartbeat-like vibration at 120 or 60 bpm, Choi and Ishii (2020) showed that the heart returned faster to a resting state after physical exercise when 60 bpm vibrations were delivered. Zhou et al. (2020) tested the effect of HR biofeedback using a vibration little box that the participant had to hold. Their results showed an increase in HRV and a reduction in HR. Ban et al. (2018) employed respiratory biofeedback using a cushion placed on the participant, which actuated at 7 or 15 bpm. They obtained an entrainment effect on BR.

Using visual modality, Hamon et al. (2018) designed a biofeedback flower that could adjust its petal colors according to HR and BR. Their preliminary results showed that the device failed to act as an ambient device and did not provide any significant results. Similarly, Yu et al. (2018) investigated the effect of a biofeedback ambient light changing according to the user's HRV. The ambient change was either toward cold hue (shades of blue) or warm hue (shades of yellow) light. Their results showed a better effect of the cold hue light to modulate the user's HRV toward a relaxed state. Cold light was rated as more relaxing and less distracting than the warm light.

#### Response Modulation: General Discussion

Response modulation is a promising strategy that is not much proposed in patents. There are many ways to modulate physiological response.

Regarding biofeedback, there is a strong link between emotions and body perception. Damasio (2006) theorized the somatic markers' hypothesis, which postulates that patterns of physiological activations linked to a particular emotion are integrated in a cortical level and impact cognitive processes. The degree of awareness of one's bodily processes is referred to as “interoceptive awareness” or “interoceptive sensitivity.” Damasio's hypothesis suggests that people with high interoceptive awareness could use this bodily information as a guidance for cognition and for emotion regulation. Indeed, studies points out that this interoceptive awareness plays a major role in regulating emotions due to the feedback loop existing between the bodily information and the emotion processing (Füstös et al., 2013). Biofeedback is precisely a technology that acts on interoceptive awareness by enhancing it (Ceunen et al., 2016), thus facilitating emotion regulation (Schoeller et al., 2019).

Entrainment seems to be a promising method. Entrainment can be defined as “the process by which independent rhythmical systems interact with each other” (Clayton, 2012). Using entrainment to elicit a particular emotion is a method well developed in the film industry through music, especially in horror movies (Winters, 2008). Several modalities can be used and physiological components be targeted to obtain an entrainment effect. Either way, studies investigated the entrainment effect mostly in the domain of music (van der Velden and Lakens, 2020), as this modality seems particularly efficient. Bergstrom et al. (2014) hypothesized that music presents the advantage to continuously draw some attention, while other modalities such as sonification does not present particular significance and, therefore, could be easily ignored by the brain. In addition, the music can by itself elicit relaxation or positive emotions.

### General Discussion

Next, we would like to give a critical view and remark on some relevant aspects of the selected contributions. First, the proportion of each regulation strategy differs substantially between patents and actual or potential papers. Even when most experimental studies focus on response modulation strategies, these strategies are overlooked in patents (Figures 6, 7), which tend to favor attentional deployment based. This fact can be a bit surprising since we would assume that patent strategies have been tested previously in experimental studies to guarantee their effectiveness. However, no recent papers were found on the same applications. Most of the *actual papers* showed the feasibility of their implementation in a controlled laboratory driving task. Arguably, the next step would be further research using ecological settings. This would allow better insight into the real impact of this method, not only on emotion regulation but also on driver distraction before proposing prototypes.

Usually, it is difficult to assess the validity of technologies described in patents, as they are not peer reviewed and are only meant to illustrate the technical feasibility of the proposed implementation. Moreover, a lack of theoretical fundamentals could be observed in some patents and in particular concerning definitions of the emotions used. This could make some propositions very expectative and original but also groundless.

While we considered that mindless attentional deployment is feasible using techniques relying on peripheral attention (such as music or lights), our sample provided limited evidence of their real effectiveness. On the other hand, several techniques that are attentionally demanding (based on cognitive change) could fit in the context of autonomous driving. Indeed, in autonomous vehicles, usual driving activity is replaced by a supervision task, which may change the stress regulation strategies involved. Either way, some original examples can be found in the literature. For instance, Paredes and Chan (2011) proposed to send an SMS message to alert the closest contacts in a user's social network if stress is detected.

When observed as a globality, some of these results may seem contradictory, and researchers on this domain are still reluctant to apply these techniques. For instance, MacLean et al. (2013) found an increase in stress when using biofeedback, showing that interoceptive awareness could also elicit further stress. Besides, most of the studies reported can be qualified as pilot studies, with a restricted number of participants and, sometimes, subjective measurements to evaluate efficiency (see Figure 8), leading to various biases, including social desirability.

Regarding sensorial modalities, haptic modalities were used mostly in studies (to induce response modulation), while patents preferred audio or visual, where more scientific evidence about their effect exists. Touch is an interesting avenue to investigate in the field of emotion regulation, as it can be implemented in various forms (MacLean, 2009). Furthermore, recent studies demonstrate the positive effects of haptic stimulation on well-being (McDaniel and Panchanathan, 2020). As for music, patents mostly use it for attentional deployment, while potential papers mostly use it for response modulation. With this aim in mind, music attributes are modified according to the state of the user, to perform biofeedback, or to generate an entrainment effect.

**Figure 9:**
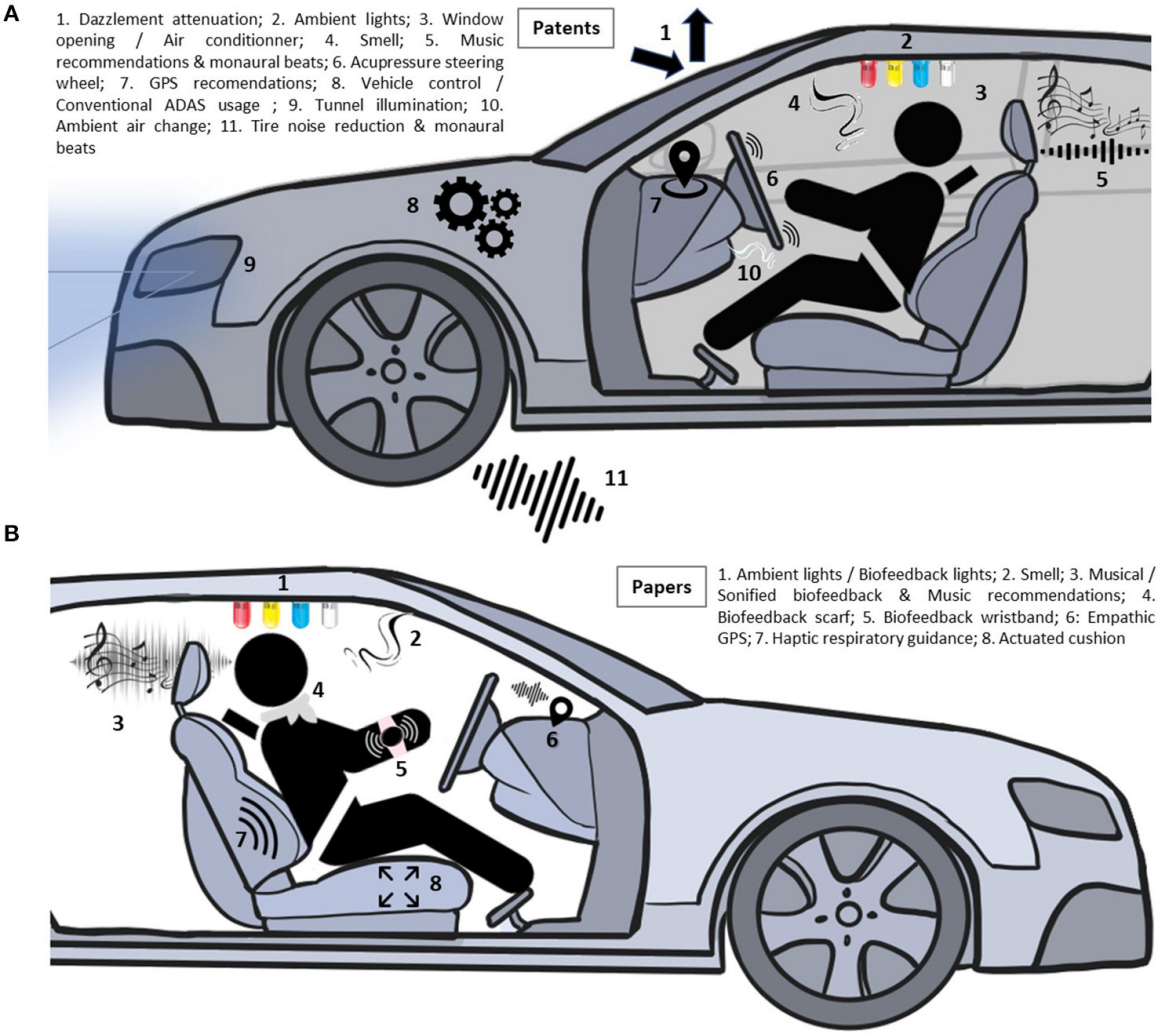
**(A)** Advanced driver-assistance systems (ADAS) implementation presented in patents and **(B)** possible ADAS according to studies.

For visual purposes, based on the present review, Figure 9A details the possible implementations of the mindless technologies tested in the studies, whereas Figure 9B displays the techniques presented in the patents. According to these figures, and considering that patents tend to implement multiple systems at once, one could wonder whether an accumulation of implementation would always be safe or disruptive. A “hysteresis effect” disrupting the driver when using multiple mindless strategies could appear. It would be relevant to investigate this point in future studies, in addition to personalization tailored to each participant, knowing that stress emergence greatly varies across individuals.

## Limitations

As any systematic review, the present work has tackled with the divergences in indexation of the studies. This issue is even more challenging in emerging fields, such as mindless computing (as defined by Adams et al., 2015), where theoretical or technological bases are not completely framed and an important list of keywords have to be used. Indeed, to be sure that few suitable papers fell out of the research, an important number of keywords was considered from the beginning, leading to an initial set of papers consisting of 1,053 results, from which <1% remained. The numerous keywords used have homonyms in very diverse topics (e.g., cellular biology or material science). This outcome can justify the use of a snowballing search strategy to guarantee an exhaustive review on the topic.

An original point of the present review is the inclusion of patents. They can present the current technologies that are being developed in the automotive industry. However, several difficulties were encountered. Most of the patent description versions in English have been translated by an automatic tool provided by Espacenet, making some definitions incomplete or hard to understand properly. Furthermore, this literature is not necessarily strict in definitions of emotions, making it sometimes difficult to disentangle between the actual emotions regulated in patents. Finally, experimental results are scarcely presented in patents, and the main outcomes in terms of efficiency or pertinence are difficult to quantify and compare to classical experimental protocols. Despite such difficulties, we encourage the inclusion of this type of contributions in reviews dealing with driving, since they present modern systems that will be incorporated with ADAS.

Concerning cognitive change, we chose to put this regulation stage aside in the discussion. We considered that the techniques collected in the present review based on this regulation stage were not mindless. As the results showed, several patents (*n* = 4) proposed this strategy, among others. Cognitive change is linked to modifying the appraisal of the situation (i.e., the way that an individual evaluates the situation). Proposed techniques suggest to prompt the driver to regulate his feelings or to drive safely using various mediums including vocal alarms (CN Patent No. 109572705A, 2019; CN Patent No. 102874259A, 2013), informative lights (CN Patent No. 110393540A, 2019), or a combination of modalities such as haptics, visual displays, and sounds (CN Patent No. 110403617A, 2019). We chose to put this regulation stage aside in the discussion. Furthermore, to prompt the user to remain calm or drive safely could, in some cases, induce even more stress (Silvia, 2002).

Regarding subjective data assessing the intervention, it is suitable to collect participant insights, feelings, and attitudes toward the intervention. However, we have to be cautious while concluding, since individual differences in populations can bias some results and no further information is provided about the participants (e.g., personality, technophobia…); also, the small sample size of some studies prevent us from generalizing conclusions. Moreover, some results may be contradictory between the subjective evaluation and physiological metrics, indicating that complex dynamics in emotion regulation have to be further explored in some specific cases.

Finally, note that, even when Gross model is a suitable approach to classify emotion regulation strategies, Gross underlined some possible overlap between strategies and specifically between situation selection and situation modification. Thus, such classification is laborious to conduct *a posteriori*, since some of the techniques investigated were not described following this approach.

## Conclusions and Perspectives

The establishment of this review allowed us to combine substantial literature published in the last 10 years about an emergent topic. Mindless stress regulation technologies can take various forms and modalities and can rely on several low cognitive processes, as we have illustrated based on the Gross model (2014). This review presented not only how such technologies could help improve safety and comfort in driving but also the technical feasibility of their implementation in a car.

The growing interest in mindless technologies to regulate emotional states should see an increase in the number of studies on this subject in the coming years. In the driving context, several studies already focus on emotional regulation (for reviews, see Chung et al., 2019; Braun et al., 2020) without mentioning the mindless aspect explicitly. Indeed, mindless regulation could apply to more emotions than stress, with the added difficulty of differentiating these emotions. This could be achieved using, for instance, machine learning. This tendency is also observed in patents that also consider other emotions (e.g., KR Patent No. 101601957B1, 2016).

While such implementations start to see the light of the day (see section 6 from Braun et al., 2020), further inspiration from mindless computing could work toward an ubiquitous perspective in parallel with the development of calm technologies (Weiser and Brown, 1996). This would ensure not only safety on the road but also a better integration of the human being into a world overwhelmed by technology and information.

## Data Availability Statement

The original contributions presented in the study are included in the article/supplementary material, further inquiries can be directed to the corresponding author/s.

## Author Contributions

AB was responsible for records collection and wrote the first draft of the manuscript. AB and AH-M organized the database, performed the statistical analysis, and wrote sections of the manuscript. All authors contributed to the conception and design of the study, revision, read, and approved the submitted version.

## Conflict of Interest

The authors declare that the research was conducted in the absence of any commercial or financial relationships that could be construed as a potential conflict of interest. The handling editor declared a past co-authorship with several of the authors AH-M and CJ.

## Footnotes

^1^https://ec.europa.eu/inea/en/horizon-2020/projects/h2020-transport/automated-road-transport/adasandme

^2^https://cordis.europa.eu/project/id/814999

^3^https://worldwide.espacenet.com/

## Appendix

### Patents

An Daeyun, Chang Dong, Seon Woo Seunghyun. 2019. Vehicle And Control Method For The Same. Korea Patent 20190103521A filed February 13, 2018 and issued September 05, 2019.

Chen Bing, Chen Wenqiang, Ding Wujun, Pan Zhijie, Zhang Fangwei, Zhao Fuquan, Zhu Zhuyang. 2013. Automobile driver emotion monitoring and automobile control system. China Patent 102874259A filed June 15, 2012 and issued January 16, 2013.

Chen Xia, Ding Hua, Fan Bin, Xin Qiang, Yin Xiaoping. 2016. Automobile atmosphere lamp control system and method based on safety. China Patent 105873281A filed May 27, 2016 and issued August 17, 2016.

Chen Yongchao, Cheng Xinlong, Gao Xiangming, Lai Jianmin, Sun Zhifu. 2019. Driver emotion detection and emotion pacifying system. China Patent 209186732U filed December 3, 2018 and issued August 02, 2019.

Choi Kab Keun, Hong Yong Pyo. 2016. Managing Apparatus And Method For Driver Based On Monaural Beats. Korea Patent 101601957B1 filed December 01, 2015 and issued March 03, 2016.

Choi Young Woo, Jung Woo Chul. 2014. Music Recommendation System For Vehicle And Method Thereof. Korea Patent 101427926B1 filed December 13, 2012 and issued August 8, 2014.

Chung Myung-Ae, Huh Chul, Jang Eun-Hye, Kim Sang-Hyeob, Park Byoung-Jun. 2014. Apparatus And Method For Controlling Emotion Of Drive. Korea Patent 20140080727A filed December 14, 2012 and issued July 01, 2014.

Diaz Emmanuelle. 2012. Method for Managing Stress Of Driver Of Motor Vehicle In Driving Situation, Involves Generating Assistance Appropriate To Situation Criticality And/Or State Of Stress, And Monitoring Assistance Allowed To Reduce Indicator Below Threshold. France Patent 2998159A1 filed November 22, 2012 and issued May 23, 2014.

Dong Fengge, Wang Guoyun, Xu Lei, Yu Xiaozhou. 2018. Intelligent atmosphere lamp control system in novel vehicle. China Patent 108628205A filed February 08, 2018 and issued October 09, 2018.

Frăţilă Ioan, Liviu Oprean Constantin, Tiţu Mihail. 2012. Full System Of Protection Against The Light Of Motor Vehicles Coming From Opposite Direction, During The Nighttime Circulation Of Motor Vehicles. Romania Patent 127332A2 filed August 09, 2010 and issued April 30, 2012.

Guo Zhiwei, Yang Feng, Zhang Jin. 2019. Driving auxiliary method and system for monitoring mental status of driver. China Patent 110403617A filed April 26, 2018 and issued November 05, 2019.

Hao Yiguo, Wang Junjie, Wang Quanbao. 2019. Driver emotion management method and device, and storage device. China Patent 109572705A filed December 11, 2018 and issued April 05, 2019.

He Junqiang, Li Bo, Lyu Huihua. 2019. Method, device and system for prompting safe driving based on driver emotional stability. China Patent 110393540A filed July 22, 2019 and issued November 01, 2019.

Huang Nengqiang. 2018. Automobile data recorder with effect of quickly emitting odor. China Patent 108109228A filed December 28, 2017 and issued June 06, 2018.

Im Hyoung Jin. 2011. Alpha Wave Occurrence Tire. Korea Patent 20110034326 filed September 28, 2009 and issued November 21, 2011.

Liu Tong, Wang Donghui, Wang Xue, Wu Xinggang, Xu Jiabin, Zhang Jindong, Zhang Kunpeng. 2019. Method for actively regulating and controlling emotion of driver by utilizing colors and sound effects. China Patent 110135355A filed May 17, 2019 and issued August 16, 2019.

Nishikawa Norimitsu, Yokota Yuji. 2011. Route Search Device. Japan Patent 2011027441A filed July 21, 2009 and issued February 10, 2011.

Sawada Kiyohiko, Yoshida Ichiro. 2016. Travel Control System For Vehicle. Japan Patent 2016052881A filed July 14, 2015 and issued April 14, 2016.

Sawada Tomonari. 2018. Vehicular Lighting Device. Japan Patent 2018020683A filed August 04, 2016 and issued February 08, 2018.

Suzuki Kenji. 2019. Comfort Providing Device In Vehicle And Vehicle Using The Same. Japan Patent 2019194046A filed May 02, 2018 and issued November 07, 2019.

Wang Meihang. 2018. Novel multifunctional steering wheel. China Patent 107953924A filed December 27, 2017 and filed April 24, 2018.
